# Reusable Glucose-Based Crown Ethers Anchored to PVC

**DOI:** 10.3390/molecules28237905

**Published:** 2023-12-02

**Authors:** Bertalan Varga, Dóra Ujj, Béla Mátravölgyi, Beáta Szolnoki, Béla Koczka, Zsolt Rapi

**Affiliations:** 1 Department of Organic Chemistry and Technology, Budapest University of Technology and Economics, 1111 Budapest, Hungary; varga.bertalan@edu.bme.hu (B.V.); matravolgyi.bela@vbk.bme.hu (B.M.);; 2 Department of Inorganic and Analytical Chemistry, Budapest University of Technology and Economics, 1111 Budapest, Hungary

**Keywords:** chiral crown ether, reusability, phase transfer catalysis, enantioselectivity, polymer support

## Abstract

The recovery and reuse of the enantioselective catalysts produced by tedious work are important not only from the perspective of green chemistry, but also from the point of view of productivity. Some of the carbohydrate-based crown ethers prepared in our research group were able to generate significant asymmetric induction in certain cases. However, they were not recoverable after the synthesis. Therefore, we modified the most effective structure with a propargyl group so that it can be attached to a polymer with an azide–alkyne reaction. It was investigated whether the position of the bonding affects the activity of the crown ethers, hence, the propargyl group was introduced either to the side chain, to the anomeric center or to the benzylidene protecting group. To anchor the macrocycles, low molecular weight PVC was modified with azide groups in 4% and 10%, respectively. It was found that glucose-based crown ether bearing the propargyl group on the benzylidene unit and grafted to PVC in 4% has the highest activity regarding the enantioselectivity (77% ee). The catalyst was recoverable in the Michael addition of diethyl acetamidomalonate to nitrostyrene and it could be reused five times without the loss of enantioselectivity.

## 1. Introduction

We live in the age of green chemistry, in which special attention is paid to catalytic methods. Valuable catalysts are used both in organocatalysis and phase transfer catalysis, which are popular nowadays [1,2,3,4,5,6]. The greatest disadvantage of most of the phase transfer processes described in the literature is that the expensive catalyst is lost after completion of the reaction; regeneration is usually not solved, or at least not economical. It is advantageous both from financial and green chemical point of view if the catalyst can be recovered and reused after the synthesis, especially if the activity and selectivity are preserved. A recoverable catalyst was used only in fraction of the large number of enantioselective phase-transfer syntheses.

The most straightforward approach to catalyst recovery is immobilization. Over the past few decades, numerous methodologies have been developed for the attachment of organocatalysts (e.g., proline, squaramide or thiourea derivatives) to various solid supports, including silica, polystyrene resin, or nanoparticles [7]. On the field of phase-transfer catalysis, several research groups bound cinchona alkaloid-based quaternary ammonium salts to polymers (e.g., to polystyrene or polyethylene glycol) [8,9,10,11,12,13,14,15,16]. In the case of the first derivatives, the alkaloid unit was directly connected to the polymer framework, while in the case of the second-generation catalysts it was recognized that if a spacer is used between the polymer and the active center of the catalyst, the selectivity can be improved. The advantage of these catalysts is that regeneration requires a simple filtration. However, their disadvantage is that the selectivity is usually reduced compared to the corresponding homogeneous analogues.

Recyclable homogeneous catalysts were elaborated to avoid the decrease in selectivity. Nájera et al. used a compound containing two cinchona units [17], which can be recovered after the completion of the reaction by precipitation with diethyl ether. A similar method was elaborated, in which a crown compound was synthesized that behaves as an ionic liquid due to its imidazolium moiety [18]. After the reactions, the organic products were selectively removed from the mixture by washing with ether. The remaining catalyst was reused after drying without a decrease in its activity.

Mauroka and his research group used a binaphthyl-based quaternary ammonium salt with perfluorinated side chains as a catalyst [19]. After the reactions, the catalyst was recovered by extraction with a perfluorinated solvent. However, the high price of the perfluorinated catalyst and solvents is a significant disadvantage of this method. The same solution was applied by Pozzi et al. In this case, the properties of a dibenzo-18-crown-6 catalyst with perfluorinated side chains were investigated [20]. The reactions were carried out in a perfluorinated solvent, then the product was extracted with an organic solvent, and the fluorinated phase was reused in the next cycle.

A special possibility to reuse the valuable phase transfer catalyst is the application of coated magnetic nanoparticles (Fe_3_O_4_). Achiral macrocycles have already been immobilized using this method. The catalysts can be recovered with the help of a magnet, other non-magnetic components of the reaction mixture can be removed by washing [21].

To the best of our knowledge, chiral crown ether catalysts were anchored to a stationary phase in only two cases. Sóti and his colleagues immobilized a glucose-based crown ether to nanofibrous silica gel [22]. The resulting catalyst was successfully applied in a Michael addition (82% ee), with a drastic increase in reaction time. Despite the possibility, the recovery of the catalyst was not carried out. Sabah et al. bound a glucose-based thiacrown ether functionalized with a propargyl group to azide-modified PVC via click reaction [23]. The resulting polymer was utilized for the extraction of various metal ions, and its capacity did not significantly change even after 10 uses. Although the immobilized macrocycle is chiral, the polymer-supported catalyst has not been used to induce enantioselectivity.

**Figure 1:**
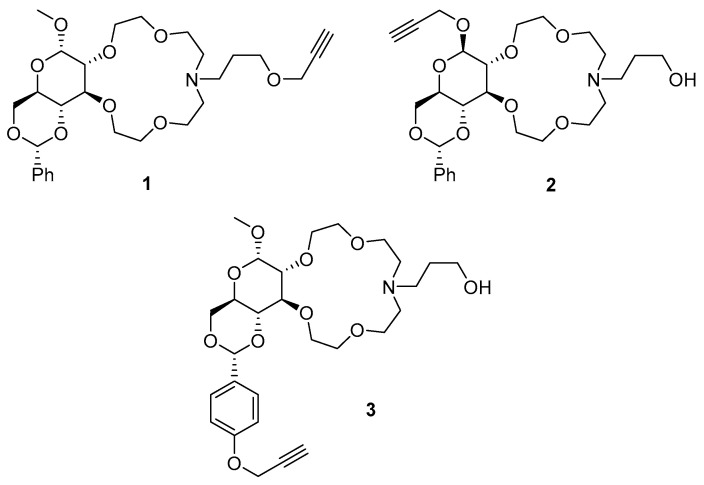
Glucose-based crown ethers (**1**–**3**) containing a propargyl group.

Herein, we report the syntheses and evaluation of a few polymer-supported glucose-based crown ethers. By modifying the monoaza-15-crown-5 structure, which proved to be the most effective in our experiments [24], three new macrocycles (**1**–**3**, Figure 1) were synthesized, which can be bound to solid carriers in different positions.

## 2. Results and Discussion

Several functional groups can be used to immobilize crown ethers. Considering the conditions of the reactions to be carried out, the azide–alkyne reaction was chosen due to the simple formation of the functional groups and the mild reaction conditions. Since the PVC selected as a carrier can be extremely easily modified with azide groups, we did not even think about its propargylation. The propargyl group was formed either on the side chain, on the anomeric center, or on the benzylidene protecting group of the crown catalyst. The choice was also confirmed by the fact that PVC-bound triazoles, which were used for the extraction of metal ions, had already been prepared [25]. Furthermore, immobilization of carbohydrates with click reaction has also been carried out [26].

### 2.1. Synthesis of Glucose-Based Crown Ethers Bearing a Propargyl Group

**Scheme 1:**
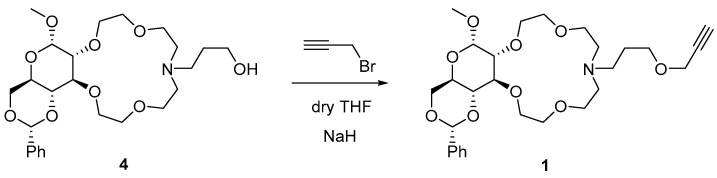
Synthesis of lariat ether **1** having a 3-(propargyloxy)propyl side arm.

Lariat ether **4**, of which synthesis is already described [27], was directly substituted with a propargyl group in its side chain (Scheme 1). For this, macrocycle **4** was reacted with propargyl bromide in THF in the presence of NaH. After column chromatography, crown compound **1** bearing a 3-(propargyloxy)propyl side arm on the nitrogen was isolated in a yield of 62%.

**Scheme 2:**
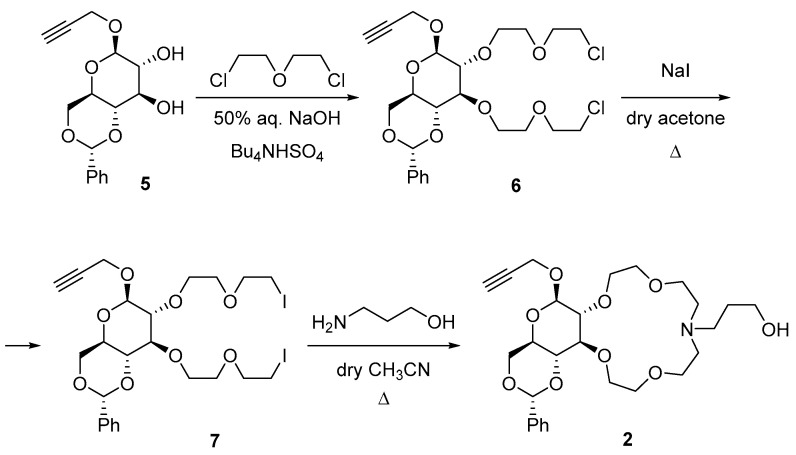
Synthesis of macrocycle **2** incorporating a propargyl β-d-glucopyranoside moiety.

The propargyl group can be introduced to the anomeric center only in the early stages of the crown ether synthesis. Propargyl-4,6-*O*-benzylidene-β-d-glucopyranoside (**5**) was synthesized according to the literature [28] and was used as the starting compound. First, it was double *O*-alkylated with bis(2-chloroethyl)ether under phase transfer conditions (Scheme 2). Bu_4_NHSO_4_ was used in equimolar amount and 50% aq. NaOH solution was the base. After the work-up procedure, bischloro derivative **6** was purified by column chromatography (28% yield). To form a better alkylating agent, compound **6** was treated with anhydrous NaI in dry acetone, which resulted in the formation of bisiodo podand **7**, and it was used without purification.

Finally, the ring closure was performed by reacting compound **7** with 3-aminopropan-1-ol in acetonitrile in the presence of sodium carbonate as depicted in Scheme 2. Macrocycle **2** containing a propargyl β-d-glucopyranoside unit was isolated in a yield of 72% after column chromatography.

**Scheme 3:**
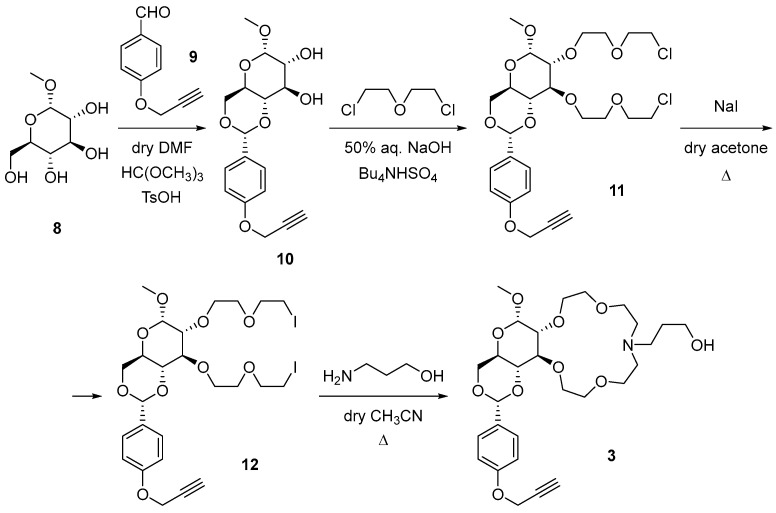
Synthesis of crown ether **3** containing a 4-(propargyloxy)benzylidene unit.

The formation of the propargyl group on the benzylidene unit is the simplest if it is already on the aromatic ring when the acetal moiety is formed. 4-Propargyloxybenzaldehyde (**9**) [29] reacted with methyl α-d-glucopyranoside (**8**) similarly to a method described in the literature [30] (Scheme 3). Trimethyl orthoformate (instead of triethyl orthoformate) was applied as a water scavenger, and the reaction was performed under vacuum to remove methanol and methyl formate formed. The desired protected glucoside **10** was obtained with moderate yield (69%) after recrystallization. Then, the previously described method was used to synthesize crown catalyst **3**. In the first step, glucose derivative **10** was *O*-alkylated with bis(2-chloroethyl)ether, then, after purification, the chlorine atoms were substituted using NaI, and finally, the reaction of bisiodo podand **12** and 3-aminopropan-1-ol yielded macrocycle **3**.

### 2.2. Immobilizing the Chiral Macrocycles on PVC

**Figure 2:**
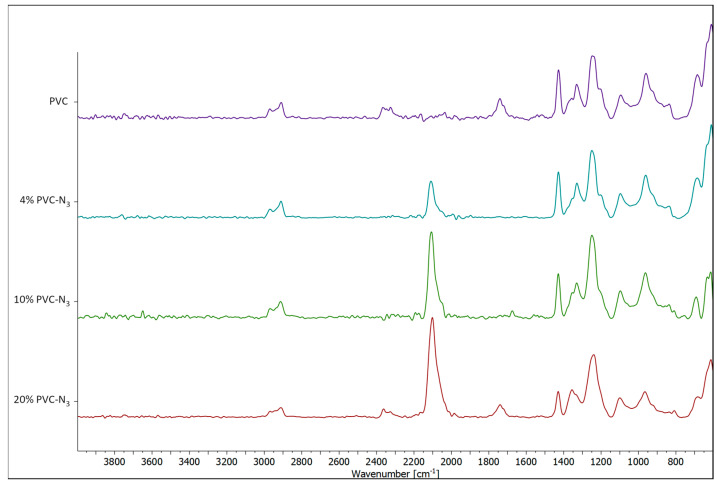
FTIR spectra of native and azide-modified PVCs (PVC-N_3_) with 4, 10, and 20% substitution.

**Scheme 4:**
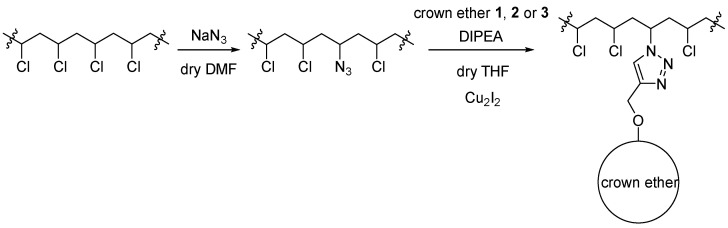
General procedure for the immobilization of the lariat ethers **1**–**3**.

Low molecular weight PVC was modified with azido groups in a nucleophile substitution according to the literature [31] in order to prepare for bonding (Scheme 4). By changing the reaction time (batches with 4% and 20% substitution) or limiting the amount of the sodium azide (batch with 10% substitution), three different batches were synthesized with approximately 4%, 10% or 20% azide substitution. The degree of the substitution was estimated by ^1^H NMR spectroscopy, since the peaks of the Cl-C*H* (appeared at 4.67–4.20 ppm) and N_3_-C*H* (appeared at 4.11–3.75 ppm) groups could be integrated separately. The FTIR spectra also showed the ascending characteristic peak of the azido group at 2102–2106 cm^−1^ (Figure 2).

**Figure 3:**
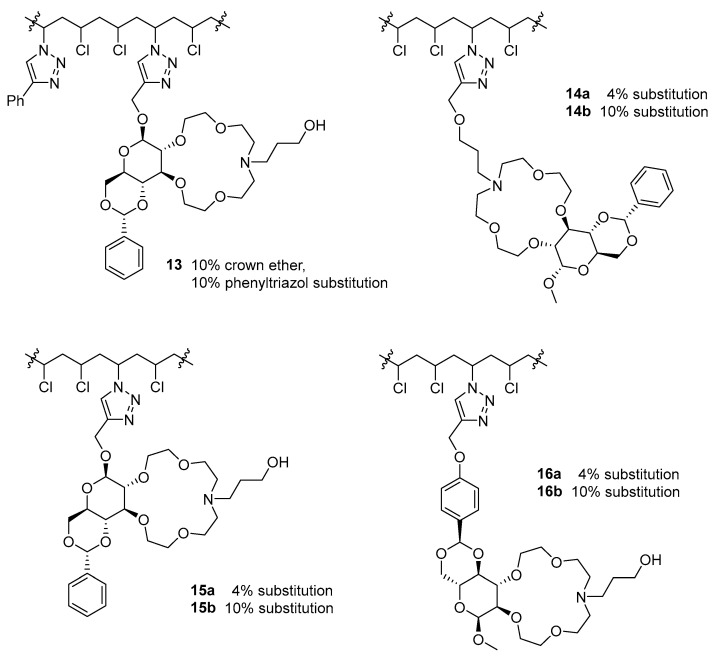
PVC-supported glucose-based crown ethers **13**–**16**.

The immobilization of the crown ethers was carried out via the azide–alkyne Huisgen reaction catalyzed by Cu_2_I_2_ and DIPEA between the azide-modified PVCs (PVC-N_3_) and the macrocycles bearing a propargyl group (**1**–**3**) (Scheme 4). After 1–2 days of stirring gelation occurred in some cases, however, dilution with dry THF yielded a low viscosity solution again, as it was before gelation. As a result of this method, seven different modified polymers were prepared (**13**–**16**) (Figure 3).

In the case of solid-supported catalyst **13**, only 10% of crown ether **2** was reacted with the 20% azide-modified PVC. After 24 h, when the crown ether was no longer detectable by TLC, phenylacetylene was added to cover the excess of the reactive azido groups. In the case of the other six polymer-supported catalysts (**14**–**16a**, **14**–**16b**), the click reactions were monitored by FTIR instead of TLC, while the reaction was carried out with an amount of crown ether corresponding to the substitution.

**Figure 4:**
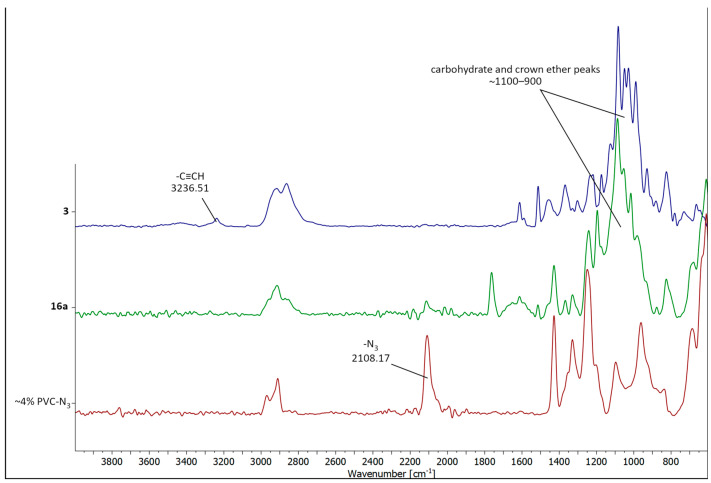
FTIR spectra of 4% PVC-N_3_, crown ether **3**, and immobilized catalyst **16a**.

The crown-modified polymers **13**–**16** were characterized by FTIR spectroscopy. As an example, infrared spectra of catalyst **16a** is shown in Figure 4. Peaks of the crown ring and the sugar moiety appear in the range of 1150–900 cm^−1^. Characteristic peaks belong to the propargyl group (3275–35 cm^−1^) and to the azide units (around 2100 cm^−1^). After the click reaction, both characteristic signals disappeared (Figure 4, middle spectra). Furthermore, the physical properties of the polymers significantly changed after the click reaction. Although, the coupling was performed in THF, the dry catalysts **13**–**16** were insoluble in THF, and in any other solvents which were tested (even at their boiling temperature): hexane, dichloromethane, diethyl ether, acetone, methyl isobutyl ketone, DMF, DMSO. For this reason, it was not possible to apply NMR spectroscopy for the characterization of the modified polymers. However, in the case of the ketones and the polar aprotic solvents, swelling in catalysts **13**–**16** was observed.

### 2.3. Examination of Different Phase Transfer Catalytic Systems

After the immobilization of crown ethers **1**–**3**, modified polymers **13**–**16** were tested as phase transfer catalysts. Three reactions were investigated in which carbohydrate-based lariat ethers were effective in previous studies [24]. The experiments were uniformly performed in a 0.5 mmol scale using 10 mol% of the non-immobilized crown ether **2** (as a reference) or the polymer-supported catalyst **13**.

**Scheme 5:**
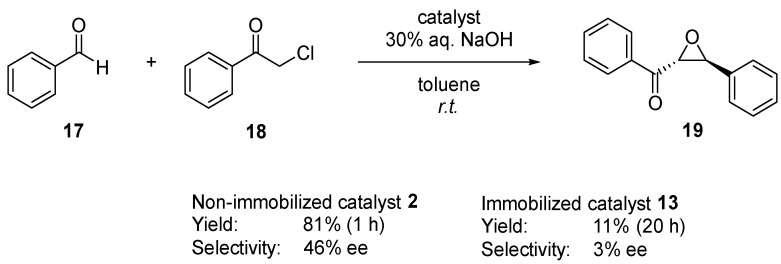
Catalyst **2** and **13** in the asymmetric Darzens condensation of benzaldehyde (**17**) and 2-chloroacetophenone (**18**).

First, PVC-supported crown ether **13** was tested in the Darzens condensation of benzaldehyde (**17**) and 2-chloroacetophenone (**18**) (Scheme 5). The reaction was carried out in toluene, while 30% aqueous NaOH solution was the base. As our previous research showed, this reaction reached full conversion after one hour if a non-immobilized lariat ether was applied [24]. However, while that was the case in the presence of crown ether **2**, when using modified polymer **13** as the catalyst, the reaction time (20 h) and even the amount of the side products highly increased. Immobilized catalyst **13** was not able to generate asymmetric induction (3% ee) or a satisfactory yield (11%). In contrast, if the Darzens reaction was carried out in the presence of compound **2**, the epoxyketone **19** was formed with a 46% enantiomeric excess and in a good yield (81%).

**Scheme 6:**
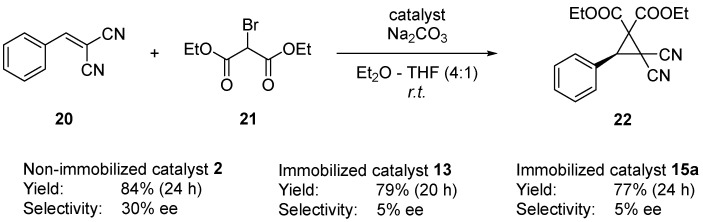
Catalyst **2**, **13** and **15a** in the asymmetric cyclopropanation of benzylidenemalonitrile (**20**) and diethyl bromomalonate (**21**).

Whereas the yield and the selectivity extremely decreased in the L/L/S system applied in the Darzens condensation (Scheme 5), a liquid-solid–solid three-phase reaction was also investigated. Benzylidenemalonitrile (**20**) reacted with diethyl bromomalonate (**21**) in a Michael-initiated ring closure (MIRC) reaction utilizing sodium carbonate as the base (Scheme 6). In the case of carbohydrate-based crown ethers it was proven previously that the enantioselectivity is the highest in a mixture of diethyl ether and THF in 4:1 ratio [24]. Despite achieving the full conversion within a similar reaction time (20–24 h), and the highly functionalized cyclopropane derivative **22** isolated with good yields (79–84%), the selectivity of the reaction strongly decreased (5% ee) when the immobilized catalyst **13** was used instead of the native crown ether **2**. One possible reason for the particularly low enantioselectivity achieved with the polymer-supported crown ether **13** could be the steric effect of the polymer chains around the chiral carbohydrate unit. Another potential reason could be the chelating effect [24] or the π–π interactions of the phenyltriazole unit, which may result in smaller energy differences in the transition states. In order to decrease the possibility of these effects, catalyst **15a**, which had a lower 4% substitution and did not contain additional phenyl groups, was employed in the MIRC reaction. However, in this case as well, a nearly racemic product (5% ee) was isolated (see Scheme 6). A phenyltriazole-bearing polymer prepared from the 20% azide-modified PVC via click reaction with excess phenylacetylene was also synthesized and tested, but it had no catalytic activity. However, the possibility of the triazole group to take part in the complexation of the cation during the reaction cannot be excluded. 

**Scheme 7:**
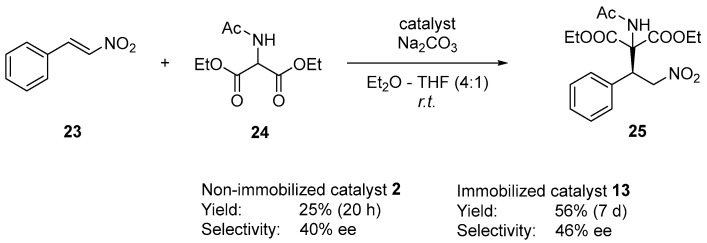
Catalyst **2** and **13** in the Michael addition of nitrostyrene (**23**) and diethyl acetamidomalonate (**24**).

Due to the good yield observed in the MIRC reaction, another similar reaction was performed. The Michael addition of nitrostyrene (**23**) and diethyl acetamidomalonate (**24**) (Scheme 7) has already been carried out in the group several times, in most cases with good enantioselectivity [24]. It was found that non-supported catalyst **2** can generate moderate asymmetric induction (40% ee), as well as its polymer-bound version, catalyst **13** (46% ee). Due to liquid-solid–solid three-phase system, the reaction time increased significantly (from 20 h to 168 h). Nevertheless, we found this reaction suitable for testing new catalysts.

### 2.4. Catalyst Evaluation

After the successful pilot asymmetric synthesis, macrocycles **1**–**3** were tested in the Michael addition of nitrostyrene (**23**) and diethyl acetamidomalonate (**24**) (see Table 1). It was found that lariat ether **3** generated the highest enantioselectivity (56% ee in 20 h, Table 1, entry 6), while crown ether **1** gave product **25** only in an ee of 18% and in twice as much time (48 h) (Table 1, entry 4). These results show that the side arm on the nitrogen has a crucial role in enantioselectivity in this reaction. The anomeric position also has an impact on the asymmetric induction, as shown in the case of macrocycle **2**, in which a propargyloxy group is attached to C-1 of the glucose unit in equatorial position. While 40% enantiomeric excess was reached using catalysts **2** (Table 1, entry 5), compound **3**, bearing an axial methoxy substituent in the anomeric center, proved to be more effective (56% ee, Table 1, entry 6). When less solvent was used in the Michael addition, complete conversion was achieved with a shorter reaction time (Table 1, entries 1–3). In the case of crown ethers **1** and **3**, the enantioselectivity increased (61% and 74%, respectively, Table 1, entries 1 and 3). Surprisingly, a higher concentration did not significantly affect the result obtained with lariat ether **2** (Table 1, entry 2). These experiments suggested that the immobilized version of compound **3**, which is catalyst **16**, is expected to be the most effective.

Next, the solid-supported catalysts **13**–**16** were tested in the Michael reaction. Comparing the results of **13** and **15b**, the presence of the phenyltriazole units decreased the enantioselectivity (46% and 56% ee, respectively, Table 2, entries 5 and 7). The trend in the effectiveness was changed in the case of **14b**, **15b** and **16b**. Despite the structural differences, the generated enantioselectivity was in the range of 44–54% (Table 2, entries 6–9). It seems that a higher substitution rate (10%) resulted in a kind of uniformization. This may be caused by the proximity of the polymer chains to the crown ring, which may affect the conformation, the stability of the complexes and the energy differences of the diastereomeric states. Catalysts **14a**, **15a** and **16a** with lower substitution rate proved to be more effective in terms of the enantiomeric excess and yield (Table 2, entries 1–4). The highest ee value was measured in the presence of **16a** (77%, Table 2, entry 3). When less solvent was used in the reaction containing catalyst **16a**, the yield and the asymmetric induction decreased (Table 2, entry 4). The reason behind this phenomenon can be that when using a higher concentration, the reaction mixture became more heterogeneous, more intense stirring was necessary and solid particles started to settle to the wall of the vessel. It is interesting that in most of the reactions, the immobilization of the crown ethers did not decrease the enantioselectivity, despite the steric effect of the polymer chain. Under the same conditions (0.10 M concentration of the Michael acceptor **23**), catalysts **14a**–**16a** and **14b**–**15b** had a higher asymmetric induction than the native lariat ethers (**1**–**3**). Moreover, compounds **15a** and **16a** were found to be more effective even compared to the 0.33 M reactions of **2** and **3**. Considering the results above, catalyst **16a** was investigated in recovery experiments. However, it is worth noting that the environment of the anomer center changed significantly in catalysts **13**, **15a** and **15b**, prepared by immobilizing crown ether **2**, which may explain the differences in their enantioselectivity. A detailed investigation of this phenomenon is planned in another work.

### 2.5. Catalyst Recovery Experiments

**Figure 5:**
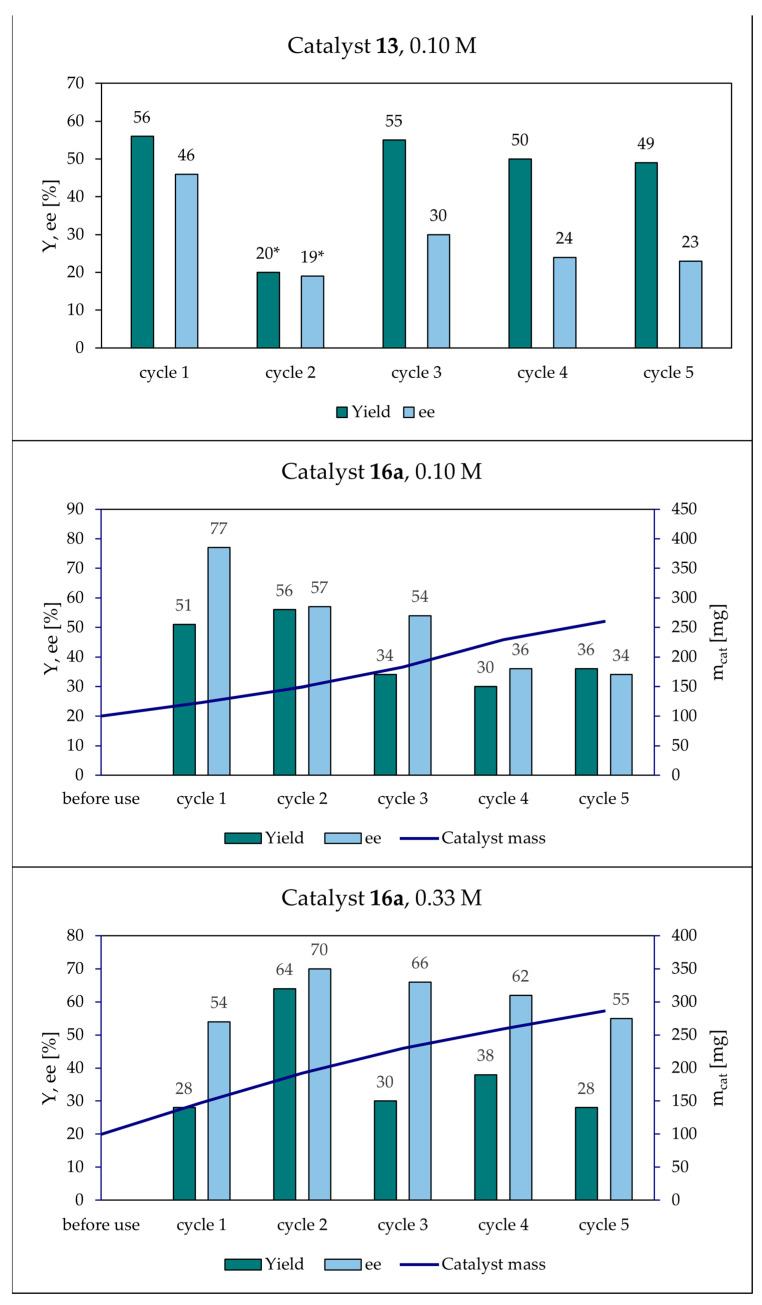
Results of the recycling experiments of **13** and **16a** (*: a part of the product was lost during preparation).

**Figure 6:**
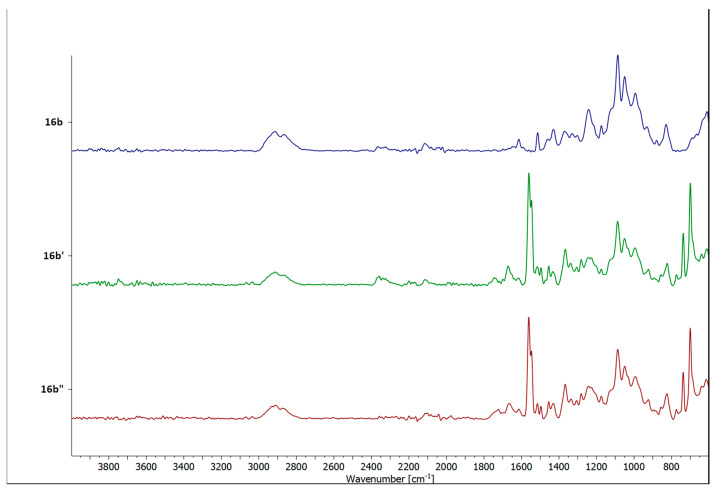
FTIR spectra of **16b** before use (blue), after reaction (**16b’**, green), and after sonication in warm dichloromethane–DMF mixture (**16b”**, red).

Recovery experiments were performed for five cycles with catalyst **13** and **16a** (Figure 5). In the latter case, two concentrations (0.1 and 0.33 M) were investigated. In each five cycles, the reaction time was constant during the experiments. In every case full conversion was achieved in the Michael addition; however, yields were variable. The reason of the lower yield was the appearance of side products, presumably from nitrostyrene as it is reported in the literature [32] and suggested by the FTIR spectra of the used catalyst (Figure 6, characteristic peak of NO_2_ group at 1560 and 1545 cm^−1^). The polymer-like side product, which was insoluble in water, acidic aqueous solutions, methanol, and even DMF, lowered the yield and increased the mass of the catalyst. It was attempted to remove the unwanted compounds from the catalyst, but even treatment with warm DMF or DMF–dichloromethane mixture while undergoing sonication [32] was not able to dissolve those. Since the polymerization of nitrostyrene is photoinduced, a Michael addition was carried out in the dark (Table 2, entry 8). In this case, a yield of **25** was better, but the formation of the unwanted side products remained significant.

Selectivity and yield decreased in each cycle, as depicted in Figure 5; the standard deviation of the yield was high. In case of catalyst **16a** used in the 0.33 M reaction mixture, the enantiomeric excess and the yield covered a wider range. The liquid-solid–solid system was too heterogeneous, and stirring was not steady; for this reason, it was hard to reproduce. The mass of the catalyst separated by filtration increased round after round.

**Figure 7:**
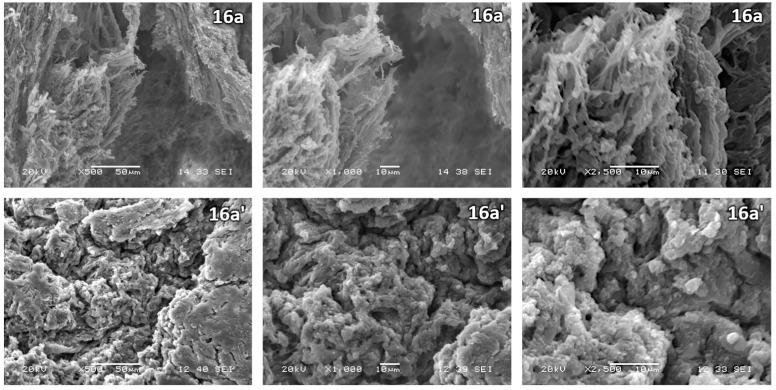
SEM microphotographs of **16a** before use and after the reaction (**16a’**); with a zoom of 500× (**left**), 1000× (**middle**), and 2500× (**right**).

Catalyst **16a** was investigated by scanning electron microscopy (SEM) (see Figure 7). The unused structure of the polymer is porous (Figure 7, **16a**), but after the first cycle, the cavities were mostly blocked (Figure 7, **16a’**). Due to the reduced porosity, the accessible surface for both organic compounds and the base was smaller. Based on the SEM analysis and the increasing mass of the catalyst, the decrease in activity and selectivity was caused by the appearance of the polymer impurity.

## 3. Conclusions

In this study, three novel glucose-based, propargyl-substituted monoaza-15-crown-5 ether (**1**–**3**) were successfully synthesized and anchored to a solid carrier. For this, macrocycles **1**–**3** were reacted with azide-modified PVC (4%, 10%, or 20% substitution) in the Huisgen-type click reaction, resulting in the formation of seven different polymer catalysts with lariat ether substitution at 4% (**14**–**16a**) or 10% (**13**, **14**–**16b**). Throughout the investigation of different phase-transfer catalytic systems, we found that **13** was effective in a three-phase Michael addition of diethyl acetamidomalonate (**24**) to nitrostyrene (**23**) (46% ee, Scheme 7). Thus, the native crown ethers (**1**–**3**) and the immobilized catalysts (**13**–**16**) were evaluated in this Michael addition (Table 1 and Table 2). The results of the reactions with macrocycles **1**–**3** showed that a higher concentration of the substrate (0.33 M instead of 0.1 M) was favorable. Furthermore, the polymers with 4% crown ether substitution generally produced higher yields and enantiomeric excesses than the catalysts with a higher substitution rate. Experimental evidence illustrates that, in most cases, the enantioselectivity of **1**–**3** did not decrease after the immobilization; the efficiency of the PVC-supported lariat ethers **15a** and **16a** was even higher than that of **2** and **3** in the reactions with a concentration of 0.33 M. To assess their practical utility, the recyclability of the polymer catalysts **13** and **16a** was investigated over five cycles, as depicted in Figure 5. During the recovery experiments, a decreasing trend was observed in the yields and enantiomeric excesses, while the mass of the catalysts was constantly growing because of a polymer-like side product. The solid contamination derived from nitrostyrene (**23**), which was observed by FTIR spectra and SEM analysis as well (Figure 6 and Figure 7), could not be removed even with sonication. Regardless, the catalyst was reusable and generated asymmetric induction in every cycle. These experiments paved the way for the design of new recoverable crown ethers. As mentioned above, the environment of the anomeric center in catalysts **13**, **15a** and **15b** has changed compared to non-immobilized crown ether **2**, which may affect the induced enantioselectivity. The investigation of the immobilization method and the type of solid support is part of our next research. 

## 4. Materials and Methods

### 4.1. General

Chemicals were purchased from Merck KGaA. (Darmsadt, Germany) and used without further purification. Analytical and preparative thin-layer chromatography (TLC) was performed on silica gel plates (60 F_254_, Merck KGaA, Darmsadt, Germany), while column chromatography was carried out using 70–230 mesh silica gel and Brockman II neutral aluminium oxide. Visualization of compounds on the TLC plates was performed with 254 nm UV light or 5 *v*/*v*% sulfuric acid/ethanol stain. Melting points were determined using a Stuart SMP10 apparatus and are uncorrected. The specific rotation was measured on a Perkin-Elmer 341LC polarimeter at 22 °C. NMR spectra were recorded on a Bruker (Billerica, MA, USA) DRX-500 or Bruker-300 instrument. IR spectra were recorded on a Bruker Tensor 37 FT-IR spectrophotometer applying an ATR sensor (4000–600 cm^−1^ range, 4 cm^−1^ resolution). The enantiomeric excess was determined on a ThermoFinnigan Surveyor liquid chromatography system using different columns. In all cases, isocratic elution was applied with a mobile phase flow rate of 0.8 mL/min. The temperature was 20 °C, and the wavelength of the detector was 222 nm. Scanning electron microphotographs were recorded on a JEOL JSM-5500LV equipment. HRMS measurements were performed on a Xevo G2-XS Q-Tof mass spectrometer, with the default ESI ionization method.

### 4.2. Methyl-4,6-O-benzylidene-2,3-dideoxy-β-d-glucopyranosido [2,3-h]-N-[3-(propargyloxy)propyl]-1,4,7,10-tetraoxa-13-azacyclopentadecane *(**1**)*

Methyl-4,6-*O*-benzylidene-2,3-dideoxy-α-d-glucopyranosido [2,3-h]-*N*-[3-hydroxypropyl]-1,4,7,10-tetraoxa-13-azacyclopentadecane (0.4 g, 0.80 mmol) (**4**) was dissolved in dry THF (30 mL), then sodium hydride (31 mg, 1.3 mmol) was added in small portions under argon. After stirring for 15 min propargyl bromide (0.100 mL, 1.3 mmol) was added dropwise. The reaction mixture was stirred at rt for 3 h, then it was quenched with 1 mL of water and was evaporated in vacuo. The residue was dissolved in dichloromethane (20 mL), then the solution was washed with water (3 × 20 mL). The combined aqueous layers were extracted with 10 mL of dichloromethane, and the combined organic phase was washed with brine (10 mL), followed by drying over Na_2_SO_4_. The solvent was removed by a rotary evaporator. The crude product was purified by column chromatography on alumina (20 g) using gradient elution (CH_2_Cl_2_–CH_3_OH 100:2 to 100:5).

Yield: 62% (0.264 g), white solid. ${\left[\alpha \right]}_{D}^{22}$ = +35.8 (*c* = 1.0, CHCl_3_). HRMS (ESI/Q-TOF) m/z: [M+H]^+^ calculated for C_28_H_42_NO_9_^+^ 536.2854, found 536.2866.

^1^H NMR (500 MHz, CDCl_3_) δ 7.49–7.43 (m, 2H, Ar*H*), 7.40–7.31 (m, 3H, Ar*H*), 5.52 (s, 1H, ArC*H*), 4.85 (d, *J* = 3.6 Hz, 1H, H-1), 4.27 (dd, *J* = 9.9, 4.6 Hz, 1H, H-6a), 4.13 (d, *J* = 2.3 Hz, 2H, C*H*_2_CCH), 3.99–3.51 (m, 18H, 7 × OC*H*_2_, H-6b, H-5 H-4, H-3), 3.47 (dd, *J* = 10.5, 5.2 Hz, 1H, H-2), 3.43 (s, 3H, OC*H*_3_), 3.01–2.50 (m, 6H, 3 × NC*H*_2_), 2.42 (t, *J* = 2.3 Hz, 1H, CC*H*), 1.93–1.68 (m, 2H, CH_2_C*H*_2_CH_2_).
^13^C NMR (126 MHz, CDCl_3_) δ 137.56, 129.08, 128.38, 126.18, 101.45, 98.53, 82.39, 80.10, 78.26, 74.37, 72.65, 70.76, 70.63, 70.17, 69.22, 62.38, 58.25, 55.36, 53.39, 29.84.
FT-IR (ATR, cm^−1^): 3263.51 (C≡CH), 2929.83, 2900.90, 2860.40, 2794.82, 1450.45, 1371.37, 1282.65, 1253.71, 1139.92, 1120.63, 1085.91, 1056.98, 1028.05, 1010.69, 991.40, 918.10, 871.81, 740.66, 692.44, 677.01, 650.00.

### 4.3. Propargyl-4,6-O-benzylidene-2,3-bis-O-[(2-chloroethoxy)ethyl]-β-d-glucopyranoside *(**6**)*

Propargyl-4,6-O-benzylidene-β-d-glucopyranoside (**5**) (9.0 g, 29.4 mmol) was dissolved in bis(2-chloroethyl) ether (70 mL, 597 mmol) in a two-necked round-bottom flask fitted with a mechanical stirrer. Tetrabutylammonium hydrogensulfate (5.0 g, 14.7 mmol) and a 50 m/m% NaOH solution (70 mL) was added to the mixture. After stirring at room temperature for 16 h, the emulsion was poured into a mixture of 150 mL dichloromethane and 150 mL water. The phases were separated, and the aqueous layer was extracted with dichloromethane (3 × 60 mL). The combined organic phase was then washed with water (2 × 60 mL), dried over Na_2_SO_4_, and concentrated under vacuum. Excess bis(2-chloroethyl) ether was removed by vacuum distillation. The crude product was roughly purified by column chromatography on silica gel (300 g) using a gradient elution (CHCl_3_–CH_3_OH 100:0 to 100:4). This was followed by a finer purification on silica gel (200 g) with hexane–EtOAc (2:1) eluent.

Yield: 28% (4.30 g), yellowish oil. ${\left[\alpha \right]}_{D}^{22}$ = -56.5 (*c* = 1.0, CHCl_3_). HRMS (ESI/Q-TOF) m/z: [M+H]^+^ calculated for C_24_H_33_O_8_Cl_23_^+^ 519.1547, found 519.1328.

^1^H NMR (500 MHz, CDCl_3_) δ 7.51–7.45 (m, 2H, Ar*H*), 7.37 (d, *J* = 6.7 Hz, 3H, Ar*H*), 5.52 (s, 1H, ArC*H*), 4.65 (d, *J* = 7.5 Hz, 1H, H-1), 4.46–4.30 (m, 3H, H-6a, OC*H*_2_CCH), 4.06–3.47 (m, 19H, H-3, H-5, H-6b, 6 × OC*H*_2_, 2 × C*H*_2_Cl), 3.43–3.36 (m, 1H, H-4), 3.31–3.24 (m, 1H, H-2), 2.48 (t, *J* = 2.5 Hz, 1H, CC*H*).
^13^C NMR (75 MHz, CDCl_3_) δ 137.29, 129.08, 128.29, 126.11, 101.62, 101.37, 82.64, 81.64, 80.77, 78.62, 77.25, 75.23, 72.46, 72.40, 71.14, 70.85, 70.81, 68.71, 66.13, 56.36, 42.81, 42.77.

### 4.4. Propargyl-4,6-O-benzylidene-2,3-bis-O-[(2-idooethoxy)ethyl]-β-d-glucopyranoside *(**7**)*

Propargyl-4,6-*O*-benzylidene-2,3-bis-*O*-[(2-chloroethoxy)ethyl]-β-d-glucopyranoside (3.20 g, 6.2 mmol) (**6**) was dissolved in dry acetone (50 mL), then dry sodium iodide (3.74 g, 24.7 mmol) was subsequently added. After refluxing for 24 h, the solvent was removed by vacuum. The residue was dissolved in dichloromethane (50 mL) followed by the filtration of the precipitate. The filtrate was washed with water (3 × 20 mL). The organic layer was then dried over Na_2_SO_4_, evaporated in vacuo, and further dried in a desiccator. The crude product was used in the next step without purification.

Yield: 93% (3.99 g), brownish oil. ${\left[\alpha \right]}_{D}^{22}$ = −40.2 (*c* = 1.0, CHCl_3_)

^1^H NMR (500 MHz, CDCl_3_) δ 7.51–7.45 (m, 2H, Ar*H*), 7.41–7.32 (m, 3H, Ar*H*), 5.53 (s, 1H, ArC*H*), 4.66 (d, *J* = 7.7 Hz, 1H, H-1), 4.42 (dd, *J* = 15.7, 2.4 Hz, 1H, C*H*_2_CCH), 4.38 (dd, *J* = 15.7, 2.4 Hz, 1H, C*H*_2_CCH), 4.33 (dd, *J* = 10.5, 5.0 Hz, 1H, H-6a), 4.04–3.86 (m, 4H, 2 × OC*H*_2_), 3.82–3.50 (m, 11H, H-3, H-5, H-6b, 4 × OC*H*_2_), 3.39 (br dt, *J* = 9.6, 4.7 Hz, 1H, H-4), 3.29–3.22 (m, 3H, H-2, C*H*_2_I), 3.18 (t, *J* = 6.9 Hz, 2H, C*H*_2_I), 2.49 (t, *J* = 2.4 Hz, 1H, CC*H*).
^13^C NMR (126 MHz, CDCl_3_) δ 137.38, 129.89, 129.14, 126.21, 101.71, 101.45, 82.74, 81.74, 80.86, 75.20, 72.57, 71.91, 71.25, 71.20, 70.89, 70.56, 70.51, 68.80, 66.23, 56.49, 3.16, 3.03.

### 4.5. Propargyl-4,6-O-benzylidene-2,3-dideoxy-β-d-glucopyranosido [2,3-h]-N-[3-hydroxypropyl]-1,4,7,10-tetraoxa-13-azacyclopentadecane *(**2**)*

Propargyl-4,6-*O*-benzylidene-2,3-bis-*O*-[(2-idooethoxy)ethyl]-β-d-glucopyranoside (3.90 g, 5.56 mmol) (**7**) was dissolved in dry acetonitrile (60 mL) under argon, then 3-aminopropan-1-ol (0.417 g, 5.56 mmol), and dry sodium carbonate (2.40 g, 22.6 mmol) was added. Following 24 h of reflux, the suspension was filtered, and the filtrate was evaporated in vacuo. The residue was then dissolved in dichloromethane (60 mL) and washed with water (3 × 30 mL), and the combined aqueous phase was extracted with dichloromethane (10 mL). The combined organic phase was dried over Na_2_SO_4_, and the solvent was removed by vacuum. The crude product was purified by column chromatography on an alumina bed (120 g) using CHCl_3_–CH_3_OH (100:0-3) as eluent.

Yield: 72% (2.08 g), white solid. Melting point: 106 °C. ${\left[\alpha \right]}_{D}^{22}$ = −64.2 (*c* = 1.0, CHCl_3_). HRMS (ESI/Q-TOF) m/z: [M+H]^+^ calculated for C_27_H_40_NO_9_^+^ 522.2698, found 522.2709.

^1^H NMR (500 MHz, CDCl_3_) δ 7.50–7.43 (m, 2H, Ar*H*), 7.40–7.31 (m, 3H, Ar*H*), 5.52 (s, 1H, ArC*H*), 4.94 (br, 1H, OH), 4.66 (d, *J* = 7.7 Hz, 1H, H-1), 4.40 (dd, *J* = 15.8, 2.5 Hz, 1H, OC*H*_2_CCH), 4.36 (dd, *J* = 15.8, 2.4 Hz, 1H, OC*H*_2_CCH), 4.32 (dd, *J* = 10.4, 4.9 Hz, 1H, H-6a), 4.02–3.89 (m, 4H, 2 × OC*H*_2_), 3.80 (t, *J* = 5.1 Hz, 2H, OC*H*_2_), 3.78–3.49 (m, 11H, H-3, H-5, H-6b, 4 × OC*H*_2_), 3.39 (td, *J* = 9.6, 4.7 Hz, 1H, H-4), 3.29–3.22 (m, 1H, H-2), 2.90–2.80 (m, 2H, NC*H*_2_), 2.74–2.66 (m, 4H, 2 × NC*H*_2_), 2.46 (t, *J* = 2.4 Hz, 1H, CC*H*), 1.75–1.60 (m, 2H, CH_2_C*H*_2_CH_2_).
^13^C NMR (126 MHz, CDCl_3_) δ 137.74, 128.96, 128.24, 126.01, 101.96, 101.13, 81.53, 80.92, 78.62, 77.23, 76.96, 75.09, 72.30, 72.17, 70.27, 70.25, 68.69, 68.65, 65.92, 64.18, 56.61, 56.31, 54.22, 54.14, 28.34.
FT-IR (ATR, cm^−1^): 3388.88 (OH), 3273.16 (C≡CH), 2933.69, 2868.11, 1450.45, 1371.37, 1352.08, 1244.07, 1176.56, 1076.26, 1043.48, 1041.55, 1008.76, 1006.83, 989.47, 972.11, 883.39, 837.09, 752.23, 692.44, 657.72.

### 4.6. Methyl-4,6-O-(4-propargyloxybenzylidene)-α-d-glucopyranoside *(**10**)*

Methyl-α-d-glucopyranoside (**8**) (15.73 g, 81 mmol) and 4-propargyloxybenzaldehyde (12.94 g, 81 mmol) (**9**) were dissolved in dry DMF (80 mL), then pTsOH monohydrate (0.385 g, 2.0 mmol), and trimethyl orthoformate (13.36 mL, 122 mmol) was added. The mixture was stirred at room temperature under reduced pressure while monitoring the reaction by TLC. After 2.5 h, an additional 4.45 mL (41 mmol) of trimethyl orthoformate was added due to incomplete conversion. Following a total reaction time of 4 h, the conversion was found to be complete. The mixture was quenched by adding 3 mL of saturated NaHCO_3_ solution to the mixture and it was subsequently concentrated in vacuum. The crude product was recrystallized from EtOAc.

Yield: 69% (18.88 g), white, solid. Mp. 187–189 °C. HRMS (ESI/Q-TOF) m/z: [M+H]^+^ calculated for C_17_H_21_O_7_^+^ 337.1282, found 337.1289.

^1^H NMR (300 MHz, CD_3_OD) δ 7.43 (d, *J* = 8.7 Hz, 2H, Ar*H*), 6.96 (d, *J* = 8.8 Hz, 2H, Ar*H*), 5.53 (s, 1H, ArC*H*), 4.76–4.68 (m, 3H, ArOC*H*_2_, H-1), 4.24–4.13 (m, 1H, H-6a), 3.81 (t, *J* = 9.3 Hz, 1H, H-3), 3.76–3.66 (m, 2H, H-5, H-6b), 3.51 (dd, *J* = 9.3, 3.8 Hz, 1H, H-2), 3.46–3.38 (m, 4H, H-4, OC*H*_3_), 2.93 (t, *J* = 2.4 Hz, 1H, CC*H*).
^13^C NMR (75 MHz, CD_3_OD) δ 158.79, 131.47, 127.94, 114.58, 102.03, 101.19, 82.00, 75.89, 73.25, 71.16, 69.13, 63.02, 55.77, 54.93, 47.86, 47.58, 47.29. The peak of the propargyl *C*H was absent.

### 4.7. Methyl-4,6-O-(4-propargyloxybenzylidene)-2,3-bis-O-[(2-chloroethoxy)ethyl]-α-d-glucopyranoside *(**11**)*

Methyl-4,6-*O*-(4-propargyloxybenzylidene)-α-d-glucopyranoside (17.0 g, 50.5 mmol) (**10**) was suspended in bis(2-chloroethyl) ether (180 mL, 1.52 mol) in a two-necked round-bottom flask fitted with a mechanical stirrer. Tetrabutylammonium hydrogensulfate (17.1 g, 50.5 mmol) and a 50 m/m% NaOH solution (180 mL) was added to the mixture. After stirring at room temperature for 16 h, the emulsion was poured into a mixture of 350 mL dichloromethane and 350 mL water. The phases were separated, and the aqueous layer was extracted with dichloromethane (3 × 120 mL). The combined organic phase was then washed with water (2 × 150 mL), dried over Na_2_SO_4_, and concentrated in vacuum. Excess bis(2-chloroethyl) ether was removed by vacuum distillation. The crude product was roughly purified by column chromatography on silica gel (600 g) using a gradient elution (CH_2_Cl_2_–CH_3_OH 100:0 to 100:6). This was followed by a finer purification on alumina (500 g) with hexane–EtOAc (2:1) eluent.

Yield: 23% (6.32 g), yellow semisolid. ${\left[\alpha \right]}_{D}^{22}$ = +45.0 (*c* = 1, CHCl_3_). HRMS (ESI/Q-TOF) m/z: [M+H]^+^ calculated for C_25_H_35_O_9_Cl_2_^+^ 549.1653, found 549.1664.

^1^H NMR (500 MHz, CDCl_3_) δ 7.42 (d, *J* = 8.4 Hz, 2H, Ar*H*), 6.96 (d, *J* = 8.5 Hz, 2H, Ar*H*), 5.48 (s, 1H, ArC*H*), 4.86 (d, *J* = 3.4 Hz, 1H, H-1), 4.69 (d, *J* = 1.9 Hz, 2H, ArOC*H*_2_), 4.25 (dd, *J* = 9.9, 4.4 Hz, 1H, H-6a), 3.98–3.47 (m, 21H, H-2, H-3, H-4, H-5, H-6b, 6 × OC*H*_2_, 2 × C*H*_2_Cl), 3.43 (m, 3H, OC*H*_3_), 2.51 (s, 1H, CC*H*).
^13^C NMR (126 MHz, CDCl_3_) δ 157.94, 130.87, 127.59, 114.74, 101.41, 99.25, 83.00, 81.80, 80.86, 79.22, 75.74, 72.39, 71.63, 71.47, 71.19, 71.01, 70.86, 69.20, 62.49, 55.96, 55.45, 43.02, 42.87.

### 4.8. Methyl-4,6-O-(4-propargyloxybenzylidene)-2,3-bis-O-[(2-idooethoxy)ethyl]-α-d-glucopyranoside *(**12**)*

Methyl-4,6-*O*-(4-propargyloxybenzylidene)-2,3-bis-*O*-[(2-chloroethoxy)ethyl]-α-d-glucopyranoside (3.36 g, 6.1 mmol) (**11**) was dissolved in dry acetone (40 mL), then dry sodium iodide (3.84 g, 26 mmol) was subsequently added. After refluxing for 48 h, the solvent was removed by a rotary evaporator. The residue was dissolved in dichloromethane (50 mL) followed by the filtration of the precipitate. The filtrate was washed with water (3 × 20 mL) and with brine (20 mL). The organic layer was then dried over Na_2_SO_4_, evaporated in vacuum, and further dried in a desiccator. The crude product was used in the next step without purification.

Yield: 86% (3.19 g), brown semisolid. ${\left[\alpha \right]}_{D}^{22}$ = + 23.5 (*c* = 1, CHCl_3_)

^1^H NMR (500 MHz, CDCl_3_) δ 7.42 (d, *J* = 8.7 Hz, 2H, Ar*H*), 6.97 (d, *J* = 8.7 Hz, 2H, Ar*H*), 5.49 (s, 1H, ArC*H*), 4.86 (d, *J* = 3.6 Hz, 1H, H-1), 4.69 (d, *J* = 2.3 Hz, 2H, ArOC*H*_2_), 4.25 (dd, *J* = 10.0, 4.6 Hz, 1H, H-6a), 3.99–3.47 (m, 17H, H-2, H-3, H-4, H-5, H-6b, 6 × OC*H*_2_), 3.43 (s, 3H, OCH_3_), 3.26 (t, *J* = 6.6 Hz, 2H, C*H*_2_I), 3.15 (t, *J* = 6.9 Hz, 2H, C*H*_2_I), 2.51 (t, *J* = 2.3 Hz, 1H, CC*H*).
^13^C NMR (126 MHz, CDCl_3_) δ 158.28, 130.95, 127.59, 114.76, 101.39, 99.26, 81.79, 80.79, 80.56, 79.26, 75.75, 72.44, 72.04, 71.87, 71.65, 70.79, 70.48, 69.19, 62.50, 55.97, 55.47, 3.19, 3.16.

### 4.9. Methyl-4,6-O-(4-propargyloxybenzylidene)-2,3-dideoxy-α-d-glucopyranosido [2,3-h]-N-[3-hydroxypropyl]-1,4,7,10-tetraoxa-13-azacyclopentadecane *(**3**)*

Methyl-4,6-*O*-(4-propargyloxybenzylidene)-2,3-bis-*O*-[(2-idooethoxy)ethyl]-α-d-glucopyranoside (1.46 g, 2.0 mmol) (**12**) was dissolved in dry acetonitrile (30 mL) under argon, then 3-aminopropan-1-ol (0.155 mL, 2.0 mmol) and dry sodium carbonate (1.27 g, 12 mmol) were added. Following 44 h of reflux, the suspension was filtered, and the filtrate was evaporated in vacuum. The residue was then dissolved in dichloromethane (40 mL) and filtered again. The filtrate was washed with water (3 × 20 mL), and the combined aqueous was extracted with dichloromethane (10 mL). The combined organic phase was dried over Na_2_SO_4_, and the solvent was removed by a rotary evaporator. The crude product was purified by column chromatography on an alumina bed (50 g) using CH_2_Cl_2_–CH_3_OH (100:5) as eluent.

Yield: 41% (0.453 g), pale semisolid. ${\left[\alpha \right]}_{D}^{22}$ = +30.3 (*c* = 1, CHCl_3_). HRMS (ESI/Q-TOF) m/z: [M+H]^+^ calculated for C_28_H_42_NO_10_^+^ 552.2803, found 552.2821.

^1^H NMR (500 MHz, CDCl_3_) δ 7.43–7.37 (m, 2H, Ar*H*), 6.99–6.93 (m, 2H, Ar*H*), 5.48 (s, 1H, ArC*H*), 4.83 (d, *J* = 3.6 Hz, 1H, H-1), 4.69 (d, *J* = 2.4 Hz, 2H, ArOC*H*_2_), 4.28–4.21 (m, 1H, H-6a), 3.96–3.51 (m, 18H, H-3, H-4, H-5, H-6b, 7 × OC*H*_2_), 3.47 (dd, *J* = 9.3, 3.6 Hz, 1H, H-2), 3.42 (s, 3H, OC*H*_3_), 2.81 (br, 6H, 3 × NC*H*_2_), 2.50 (t, *J* = 2.4 Hz, 1H, CC*H*), 1.72 (s, 2H, CH_2_C*H*_2_CH_2_).
^13^C NMR (126 MHz, CDCl_3_) δ 158.10, 130.98, 127.53, 114.73, 101.27, 98.55, 82.34, 79.96, 78.55, 78.23, 75.73, 72.65, 70.65, 70.03, 69.16, 62.36, 55.96, 55.34, 54.66, 54.37, 29.84. The peak of the propargyl *C*H was absent, and some of the O*C*H_2_ peaks were not separated.
FT-IR (ATR, cm^−1^): 3435.18 (OH), 3238.44 (C≡CH), 2916.33, 2864.25, 1612.47, 1512.17, 1454.31, 1369.44, 1303.86, 1217.07, 1172.70, 1124.48, 1083.98, 1049.26, 1029.97, 989.47, 929.68, 881.46, 823.59, 779.23, 732.94, 665.43.

### 4.10. General Method for Immobilization of Crown Ethers on PVC

PVC-N_3_ (0.20 g, 4% or 10% substitution) and a crown ether bearing a propargyl group (0.20 mmol or 0.35 mmol, respectively) were dissolved in dry THF (20 mL), then DIPEA (1.3 equiv. for the crown ether) and Cu_2_I_2_ (0.05 equiv. for the crown ether) were added. The progression of the immobilization was monitored by FTIR spectroscopy. When the characteristic peak of the azide group (around 2100 cm^−1^) was no longer detectable in a small sample (typically after 2–3 days), the reaction mixture was poured into a 3:1 water–methanol mixture. After 1 h of stirring, the entire amount of the polymer precipitated. The precipitate was alternately washed with water and methanol (4 times with each solvent) while being shredded into smaller pieces. Finally, it was dried in a desiccator to obtain a greenish elastic solid.

In the case of **13**, phenylacetylene (5.0 equiv. for the crown ether) was added after one day when the crown ether was no longer detectable by TLC, then the mixture was stirred for an additional 2 days before the standard workup procedure.

### 4.11. General Method for the Asymmetric Darzens Condensation

2-chloroacetophenone (0.077 g, 0.5 mmol) (**18**) was dissolved in toluene (3 mL), then benzaldehyde (0.071 mL, 0.7 mmol) (**17**), the catalyst (0.026 g, 0.05 mmol of **2**, or containing 0.1 equiv. of the immobilized crown ether **13**), and 30% aqueous NaOH solution (0.5 mL) were added, respectively. The mixture was stirred vigorously at room temperature, and the reaction was monitored by TLC (hexane–ethyl acetate 4:1). After completion, the catalyst was filtered off (in the case of **13**), and the filtrate was diluted with toluene (10 mL). The phases were separated, and the organic layer was washed with 10% aqueous HCl solution (3 × 10 mL). After drying over Na_2_SO_4_ and Na_2_CO_3_, the organic phase was evaporated in vacuum. The crude product was purified by preparative TLC (hexane–ethyl acetate 10:1) to give **19** as a yellowish-white solid having an mp of 64–66 °C. For the respective yields and ee values, see Scheme 5. Chiral HPLC: Phenomenex Lux^®^ 5µm Amylose-1 (LC Column 250 × 4.6 mm), CH_3_CN–H_2_O + 0.1% AcOH (70:30). For the details see the Supplementary Materials.

### 4.12. General Method for the Asymetric MIRC Reaction

Benzylidenemalonitrile (0.077 g, 0.5 mmol) (**20**) was dissolved in a 4:1 mixture of dry diethyl ether and dry THF (5 mL), then diethyl bromomalonate (0.125 mL, 0.75 mmol) (**21**), the catalyst (0.026 g, 0.05 mmol of **2**, or containing 0.1 equiv. of the immobilized crown ether **13** or **15a**), and dry sodium carbonate (0.11 g) were added, respectively. The mixture was stirred vigorously at room temperature, and the reaction was monitored by TLC (hexane–ethyl acetate 4:1). After completion, the solids were filtered off and washed with ether (3 × 5 mL), then the filtrate was evaporated in vacuo. The crude product was purified by preparative TLC (hexane–ethyl acetate 5:1) to give **22** as a yellow oil. For the respective yields and ee values, see Scheme 6. Chiral HPLC: Phenomenex Lux^®^ 5 µm Amylose-1 (LC Column 250 × 4.6 mm), CH_3_CN–H_2_O + 0.1% AcOH (50:50). For the details see the Supplementary Materials.

### 4.13. General Method for the Asymmetric Michael-Addition

Trans-β-nitrostyrene (0.075 g, 0.5 mmol) (**23**) and diethyl-acetamidomalonate (0.152 g, 0.7 mmol) (**24**) were dissolved in a 4:1 mixture of dry diethyl ether and dry THF (5 mL, or 1.5 mL in the case of Table 1, Entry 1–3 and Table 2, Entry 4), then a catalyst (0.05 mmol, or containing 0.1 equiv. of the immobilized crown ether) and dry sodium carbonate (0.11 g, 1.0 mmol) were added. The mixture was stirred vigorously at room temperature, and the reaction was monitored by TLC (hexane–ethyl acetate 4:1). After completion, the solids were filtrated and washed with ether (3 × 5 mL), then the filtrate was evaporated in vacuo. The crude product was purified by preparative TLC (hexane–ethyl acetate 3:1) to give **25** as an off-white solid having an mp of 135–136 °C. For the respective yields and ee values, see Scheme 7, Table 1 and Table 2. Chiral HPLC: Phenomenex Lux^®^ 5µm Cellulose-4 (LC Column 250 × 4.6 mm), CH_3_CN–H_2_O + 1% AcOH (45:55). For the details see the Supplementary Materials.

For the purification of the immobilized catalysts, the filtered residue was washed with water (4 × 5 mL) and methanol (2 × 5 mL), then the remaining solids were dried in a desiccator. In the case of the reactions in Table 2, entry 4, an additional washing was performed with DMF–CH_2_Cl_2_ (1:1, 2 × 5 mL) and with CH_2_Cl_2_ (1 × 5 mL).

## Figures and Tables

**Table 1:** Effect of the non-immobilized crown ethers **1–3** and the concentration in the asymmetric Michael addition of nitrostyrene (**23**) and diethyl acetamidomalonate (**24**).

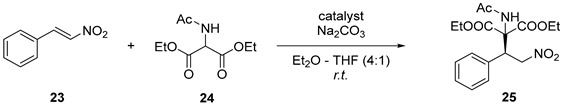					
Entry	Crown Ether	Concentration, M ^a^	Reaction Time, h	Yield, % ^b^	ee, % ^c^
1	**1**	0.33	22	41	61
2	**2**	0.33	4	37	37
3	**3**	0.33	7	72	74
4	**1**	0.10	48	30	18
5	**2**	0.10	20	25	40
6	**3**	0.10	20	37	56

^a^ Calculated for the nitrostyrene (**23**); ^b^ Isolated by preparative TLC; ^c^ Determined by chiral HPLC.

**Table 2:** The effect of the catalyst **13**–**16** in the Michael addition of nitrostyrene (**23**) and diethyl acetamidomalonate (**24**).

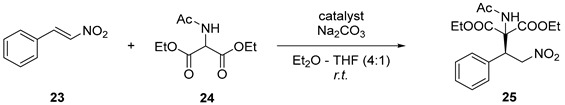				
Entry	Immobilized Crown Ether	Reaction Time, d	Yield, % ^a^	ee, % ^b^
1	**14a** (4% substitution)	13	47	40
2	**15a** (4% substitution)	5	66	58
3	**16a** (4% substitution)	2	51	77
4 ^c^	**16a** (4% substitution)	1	28	70 (54) ^d^
5	**13** (10% substitution)	7	56	46
6	**14b** (10% substitution)	2	31	44
7	**15b** (10% substitution)	1	22	54
8 ^e^	**15b** (10% substitution)	1	41	39
9	**16b** (10% substitution)	2	30	48

^a^ Isolated by preparative TLC; ^b^ Determined by chiral HPLC; ^c^ In 0.33 M concentration calculated for the nitrostyrene (**23**); ^d^ Highest selectivity in the recycling experiments (ee after the first cycle in parentheses); ^e^ The reaction was carried out in the dark.

## Data Availability

The data presented in this study are available on request from the corresponding author.

## Supplementary Materials

The following supporting information can be downloaded at: https://www.mdpi.com/article/10.3390/molecules28237905/s1., Figure S1–S6: NMR spectra of crown ethers **1**–**3;** Figure S7–S16: FTIR spectra of crown ethers **1**–**3** and immobilized catalysts **13**, **14a-b**, **15a-b**, and **16a-b;** Figure S17–S48: Chiral HPLC chromatograms of **19**, **22**, and **25.**
