# Supplementary material for: Reusable Glucose-Based Crown Ethers Anchored to PVC
Source: Molecules. 2023 Dec 2;28(23):7905. doi: 10.3390/molecules28237905 (PMC10707916; doi:10.3390/molecules28237905)
Supplement: Supplementary file 1 [file molecules-28-07905-s001.zip › molecules-2741946-supplementary.pdf]

Supplementary Information

Reusable glucose-based crown ethers anchored to PVC

Bertalan Varga [1], Dóra Ujj [1], Béla Mátravölgyi [1], Beáta Szolnoki [1], Béla Koczka [2] and Zsolt Rapi [1,*]

[1] Department of Organic Chemistry and Technology, Budapest University of Technology and Economics, Budapest, 1111, Hungary
[2] Department of Inorganic and Analytical Chemistry, Budapest University of Technology and Economics, Budapest, 1111, Hungary
* Correspondence: rapi.zsolt@vbk.bme.hu

Table of contents Page

– NMR spectra of crown ethers **1-3** S1-S6

– FTIR spectra of crown ethers **1-3** and immobilized catalysts **13**, **14a-b**, **15a-b**, and **16a-b** S7-S16

– Chiral HPLC chromatograms of **19**, **22**, and **25** S17-S48

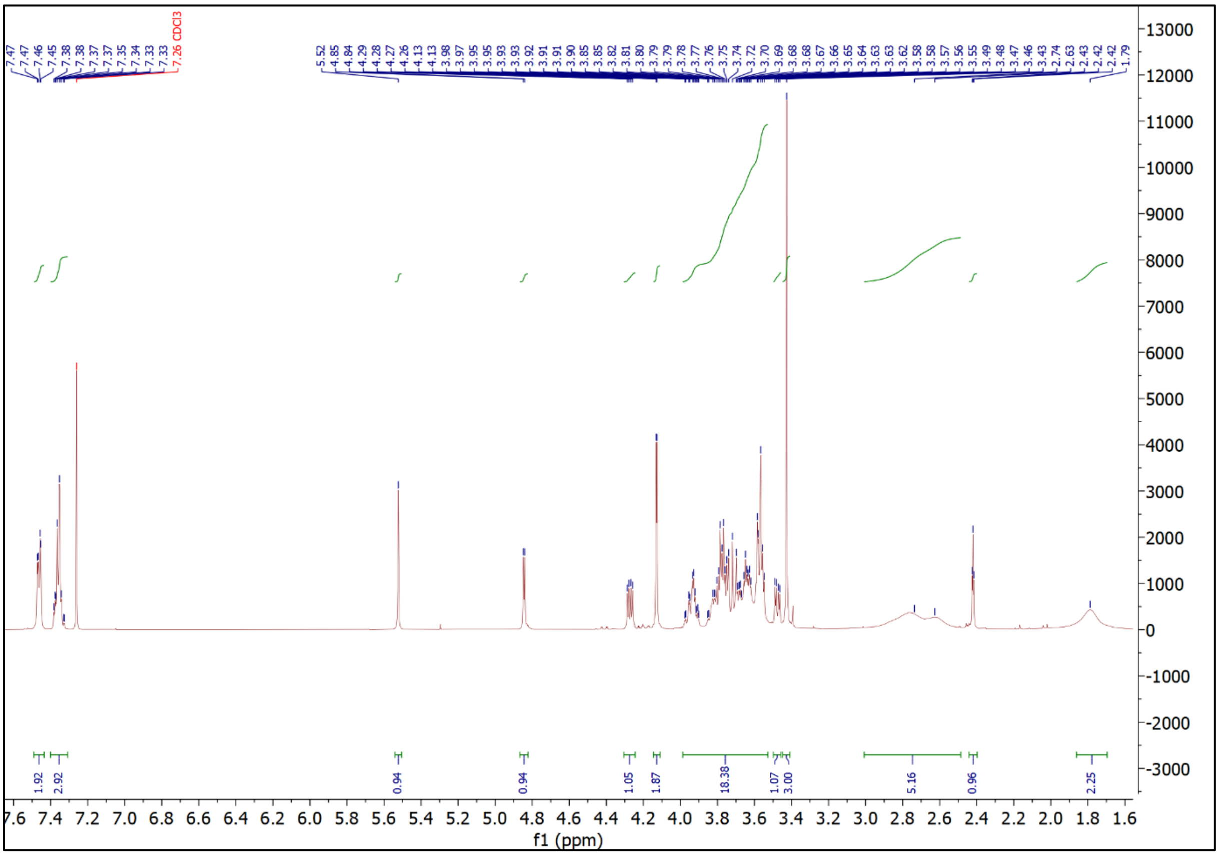

Figure S1. ^{1}H NMR spectra of crown ether **1**.

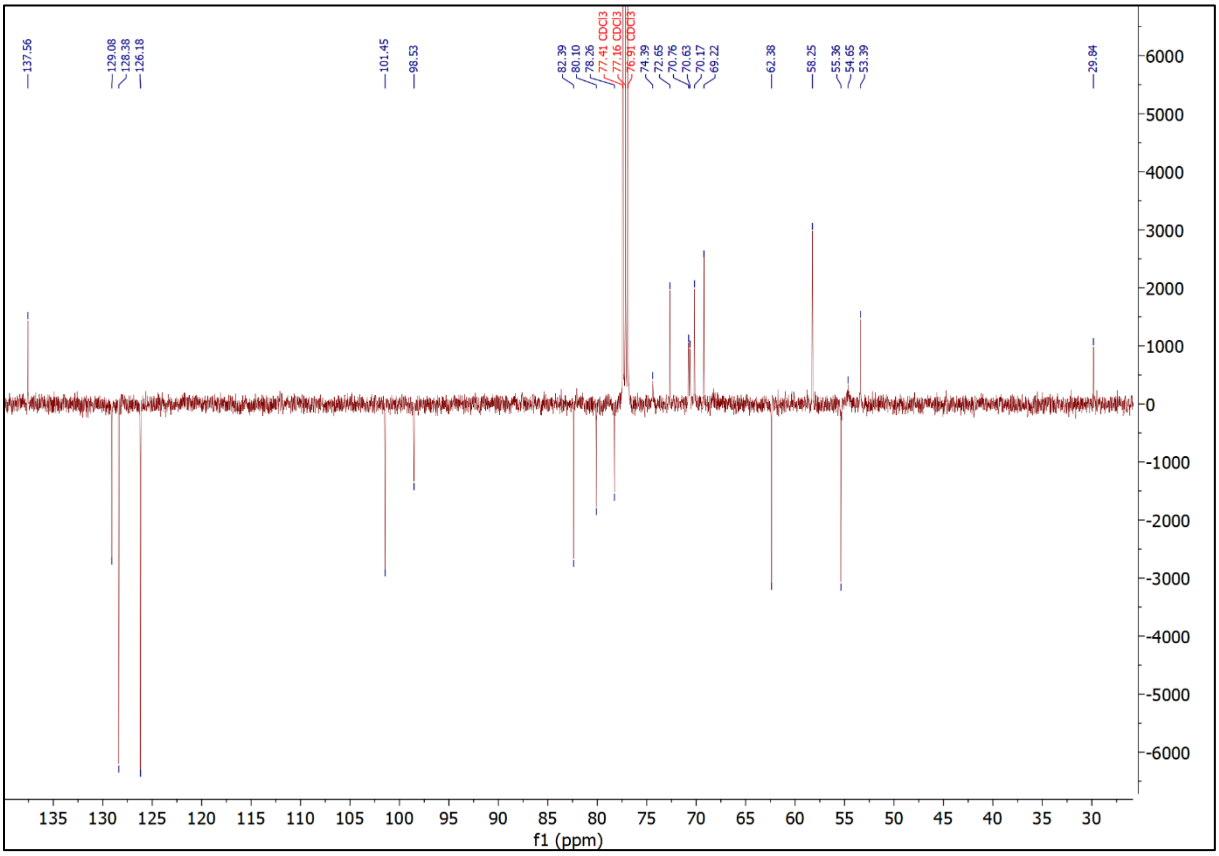

Figure S2. ^{13}C NMR (APT) spectra of crown ether **1**.

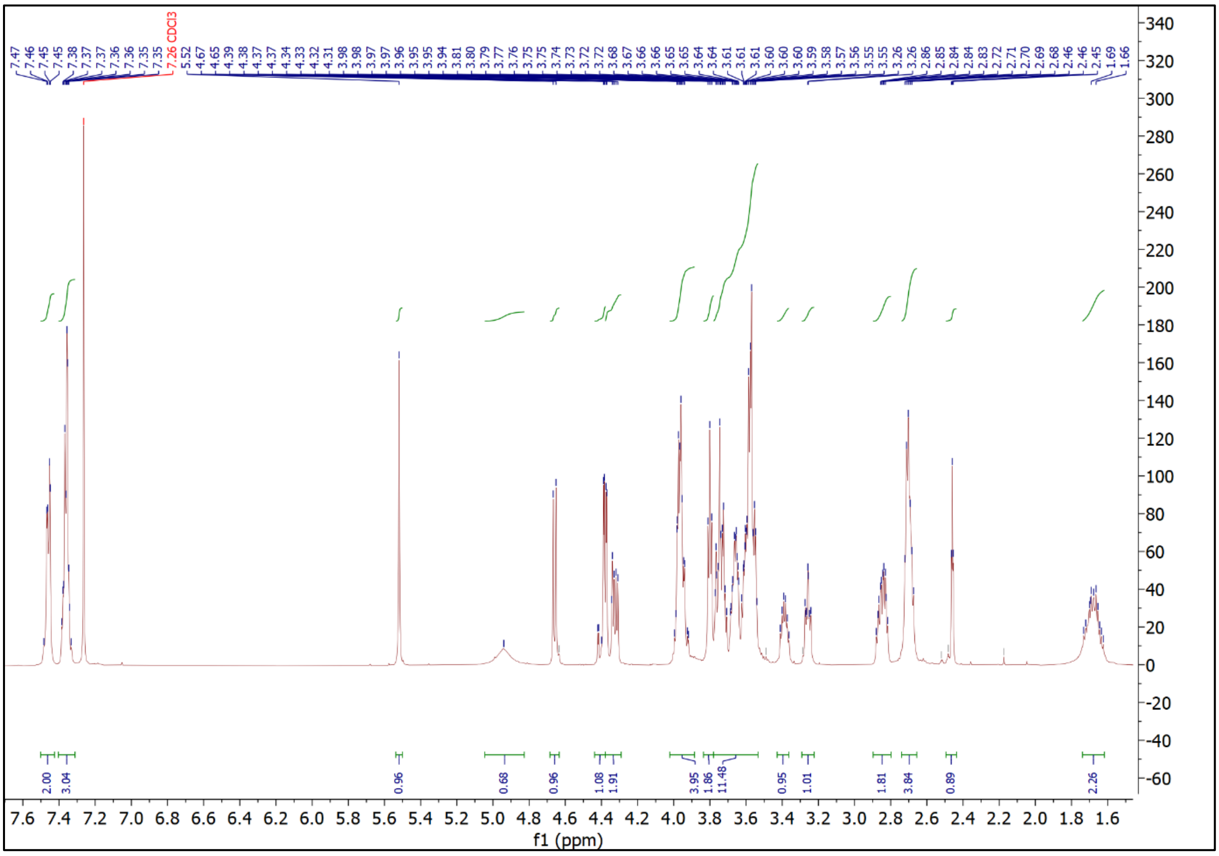

Figure S3. ^{1}H NMR spectra of crown ether **2**.

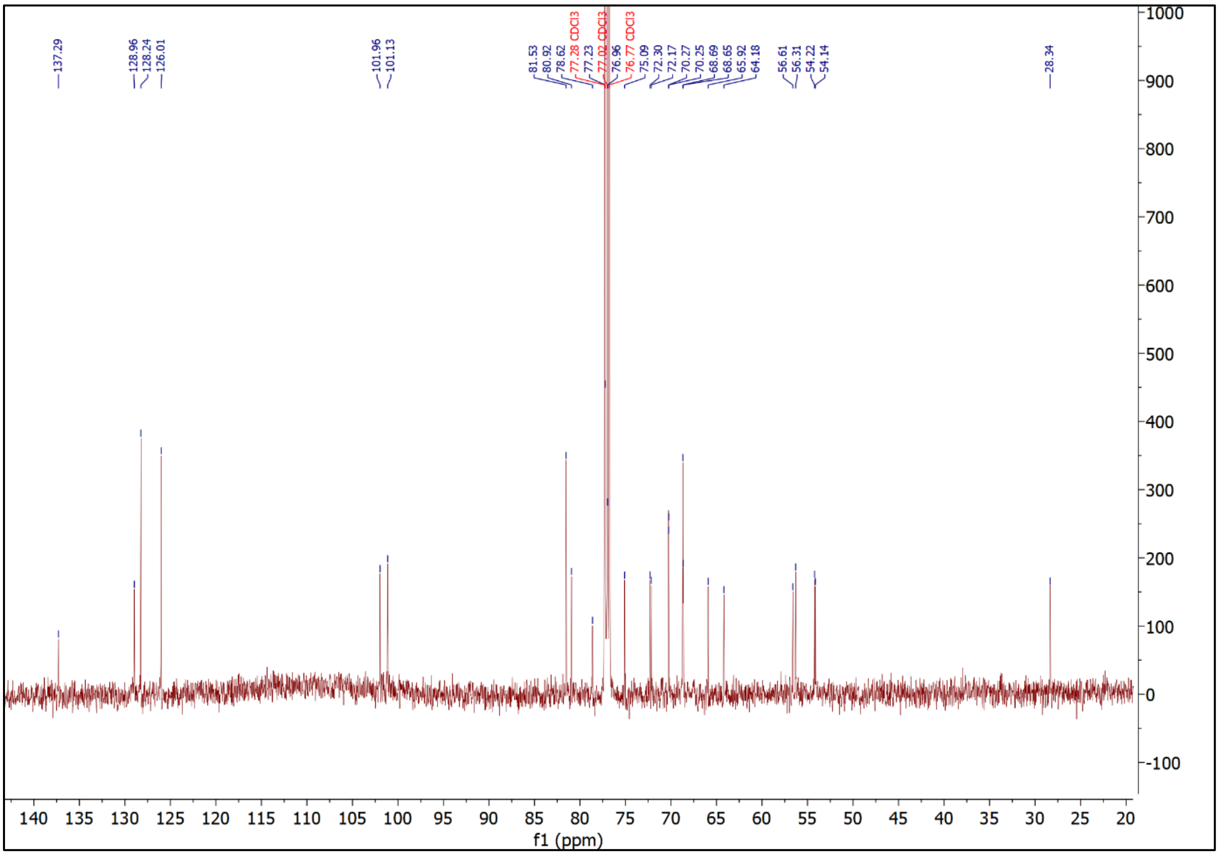

Figure S4. ^{13}C NMR (APT) spectra of crown ether **2**.

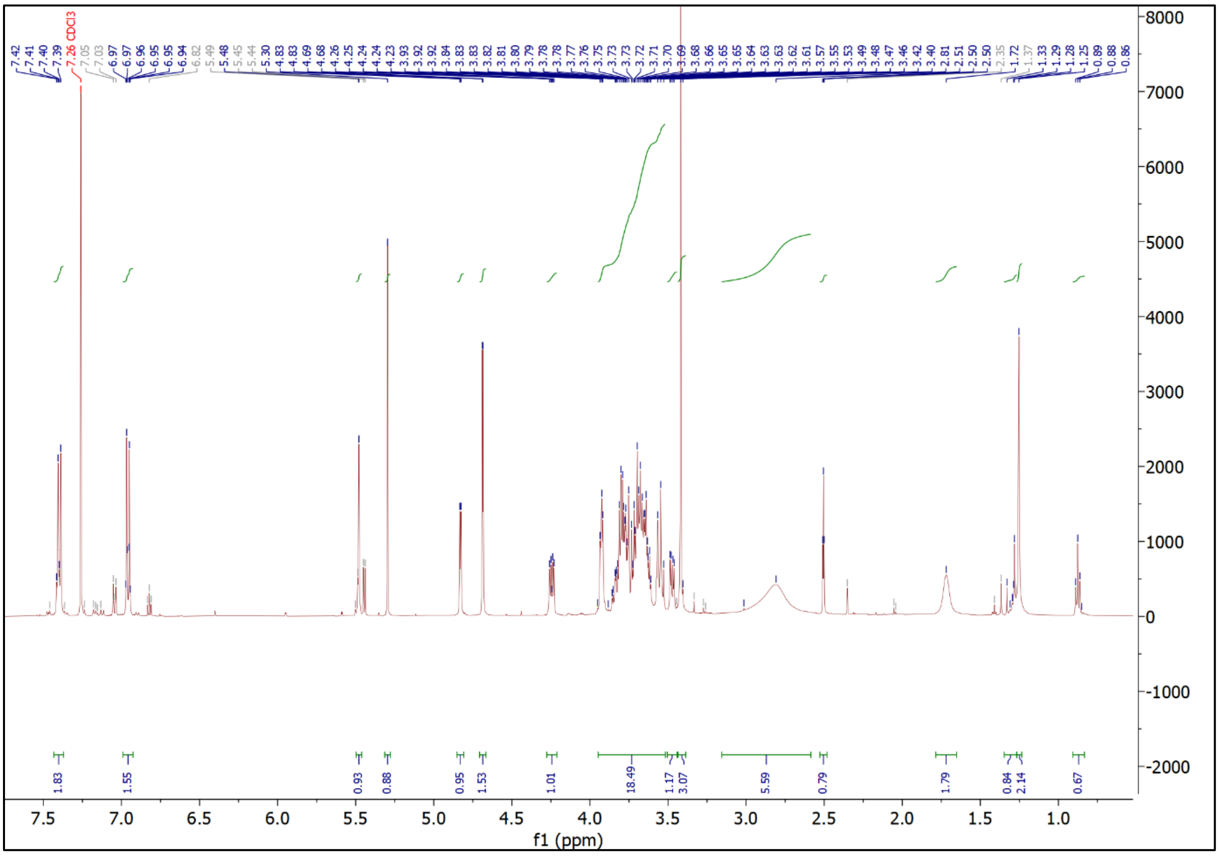

Figure S5. ^{1}H NMR spectra of crown ether **3**.

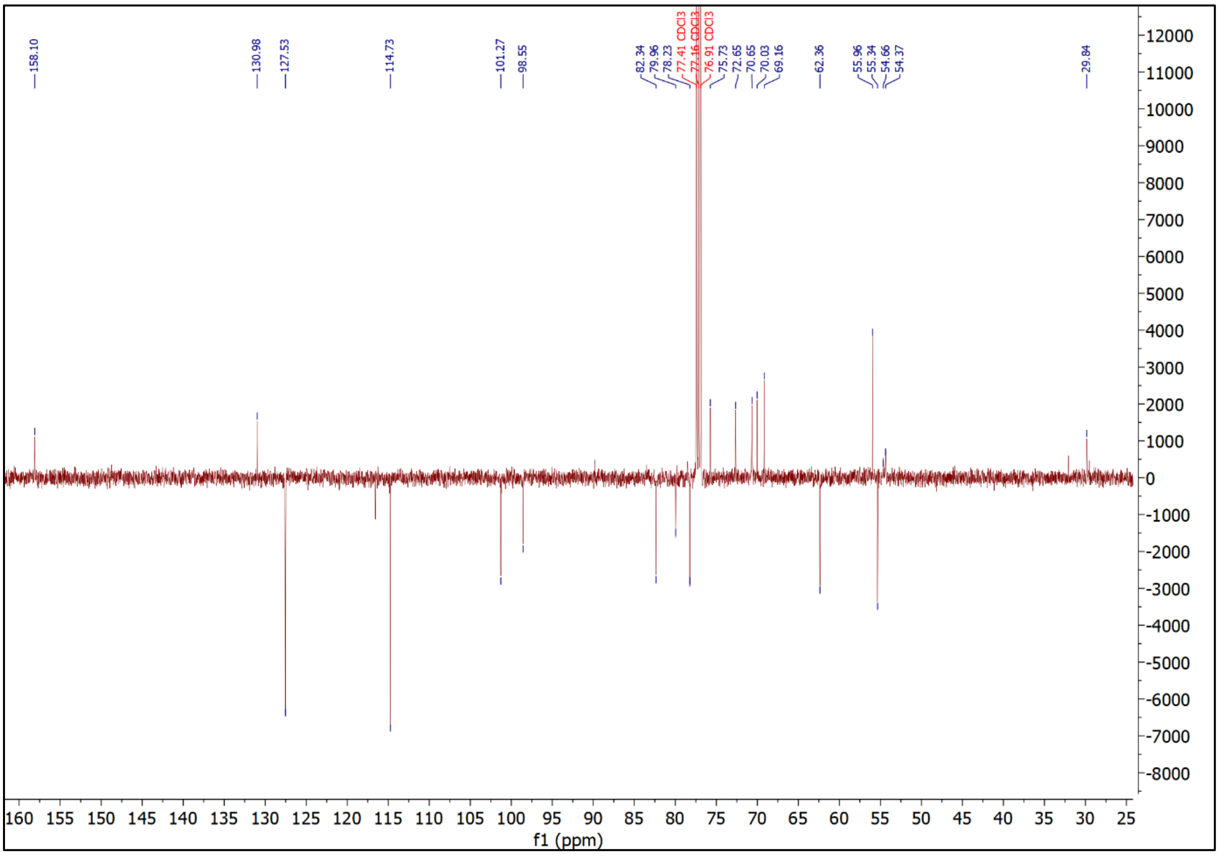

Figure S6. ^{13}C NMR (APT) spectra of crown ether **3**.

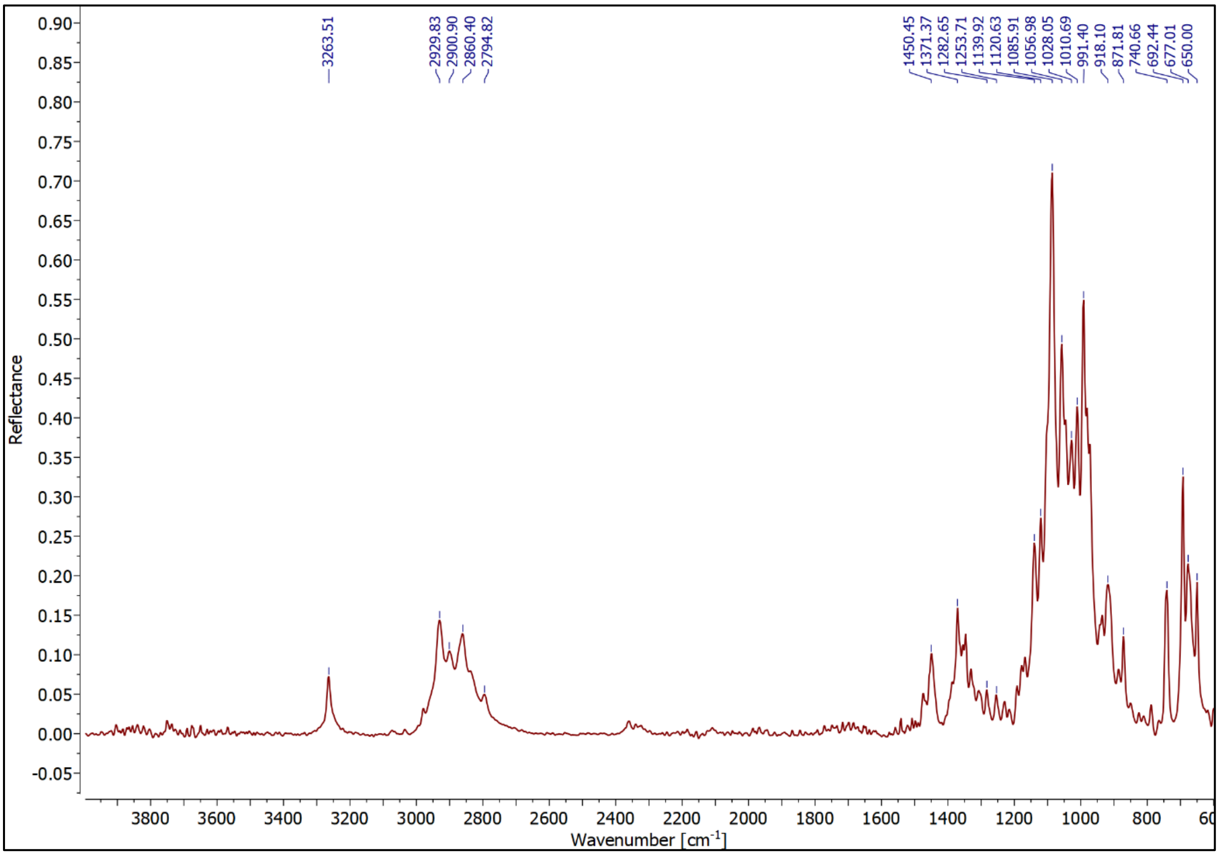

Figure S7. FTIR (ATR) spectra of crown ether **1**.

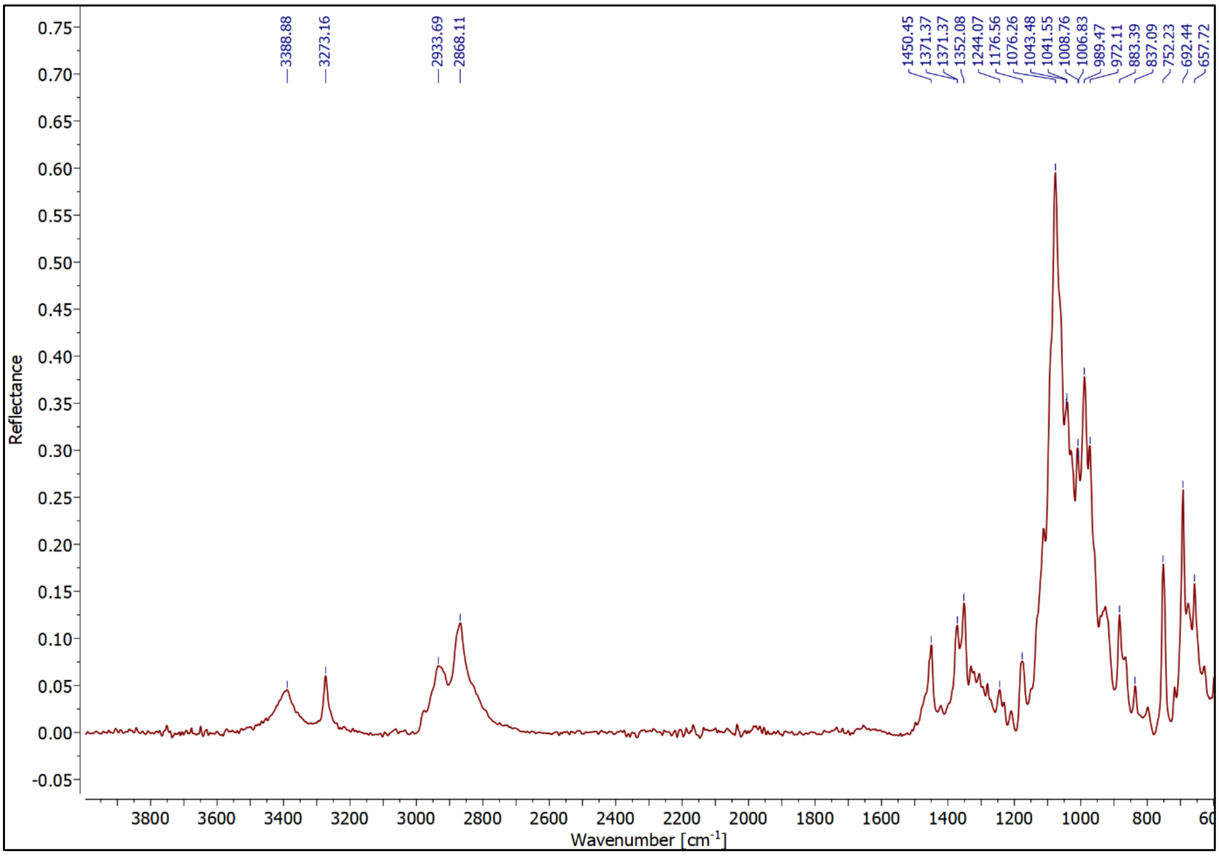

Figure S8. FTIR (ATR) spectra of crown ether **2**.

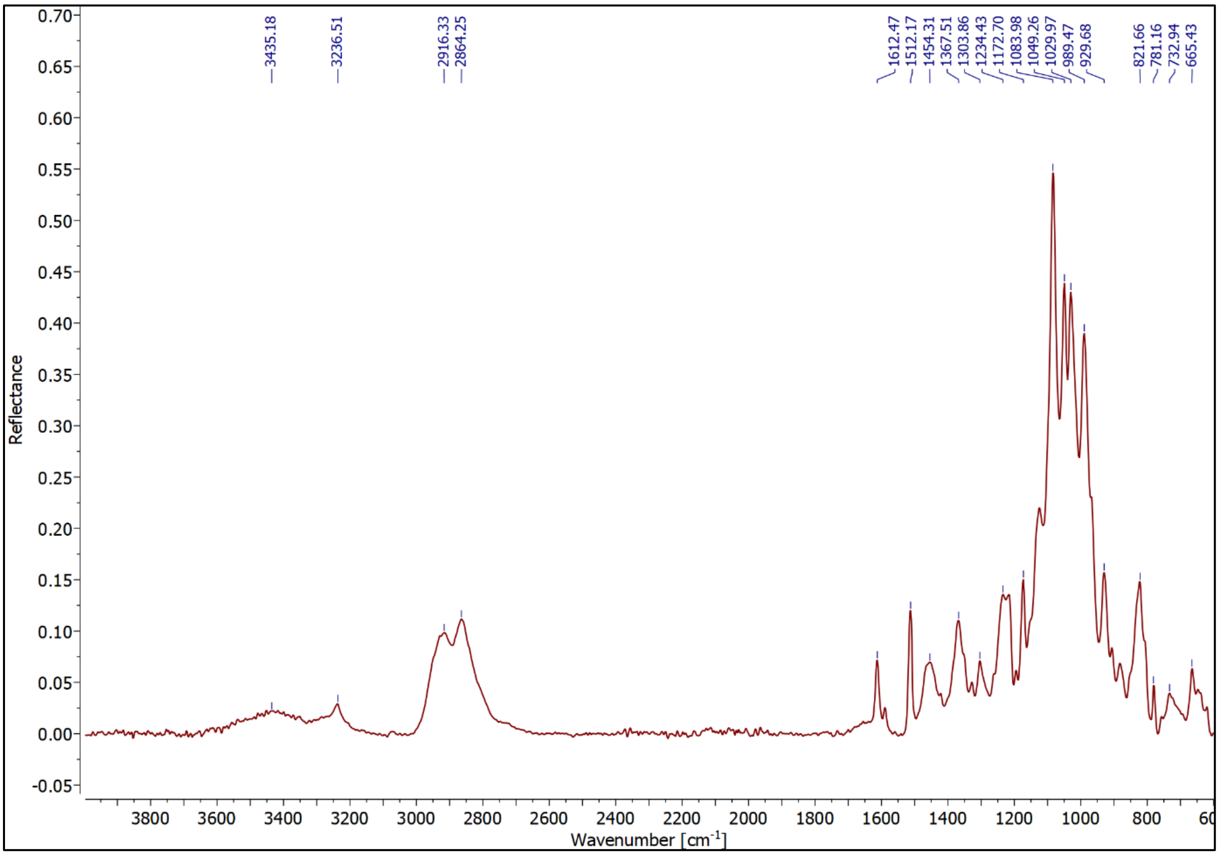

Figure S9. FTIR (ATR) spectra of crown ether **3**.

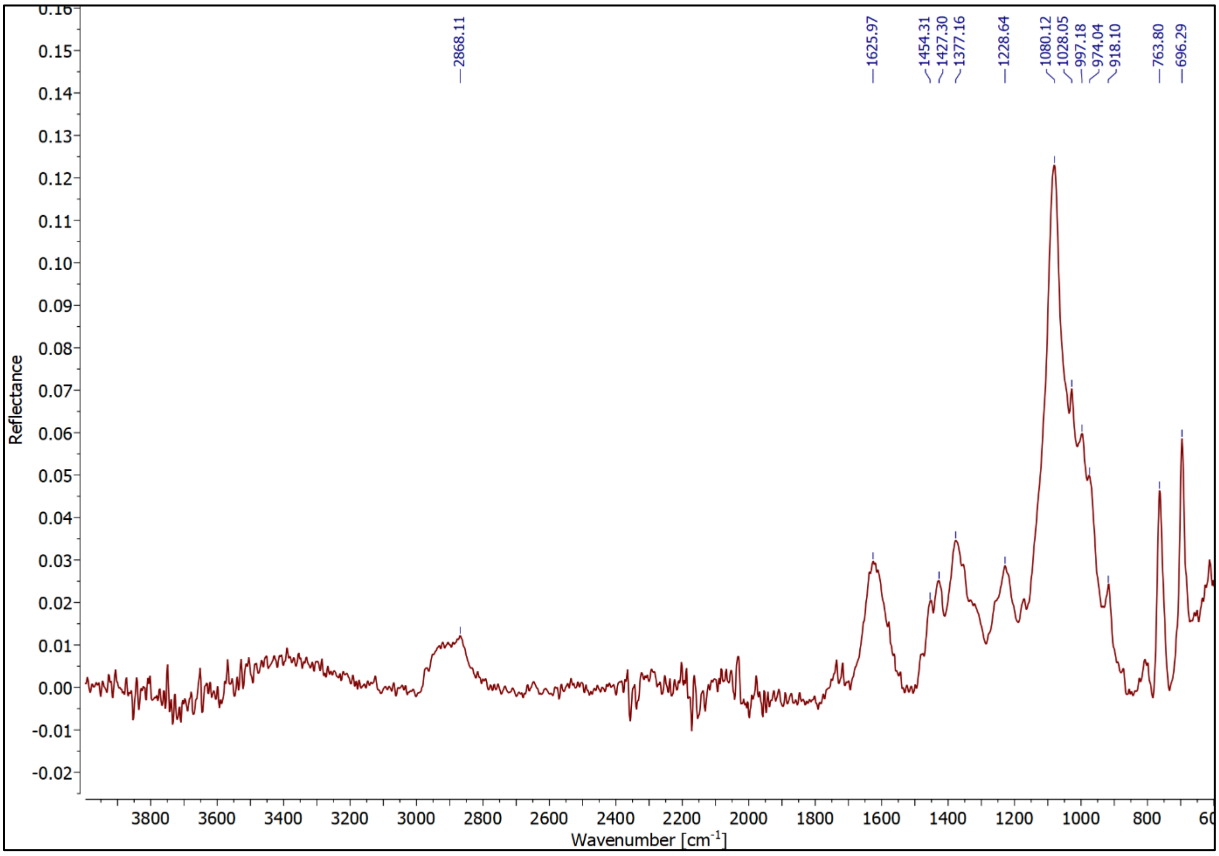

Figure S10. FTIR (ATR) spectra of immobilized catalyst **13**.

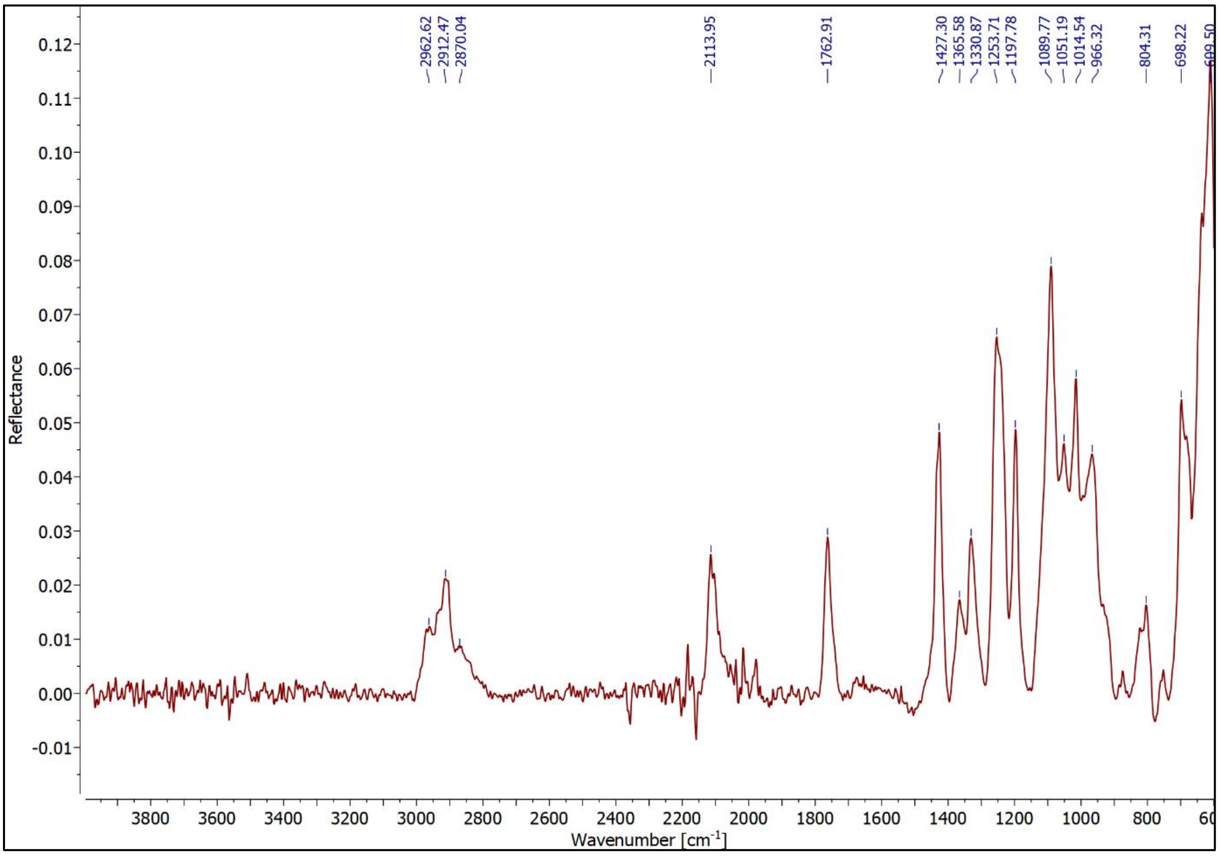

Figure S11. FTIR (ATR) spectra of immobilized catalyst **14a**.

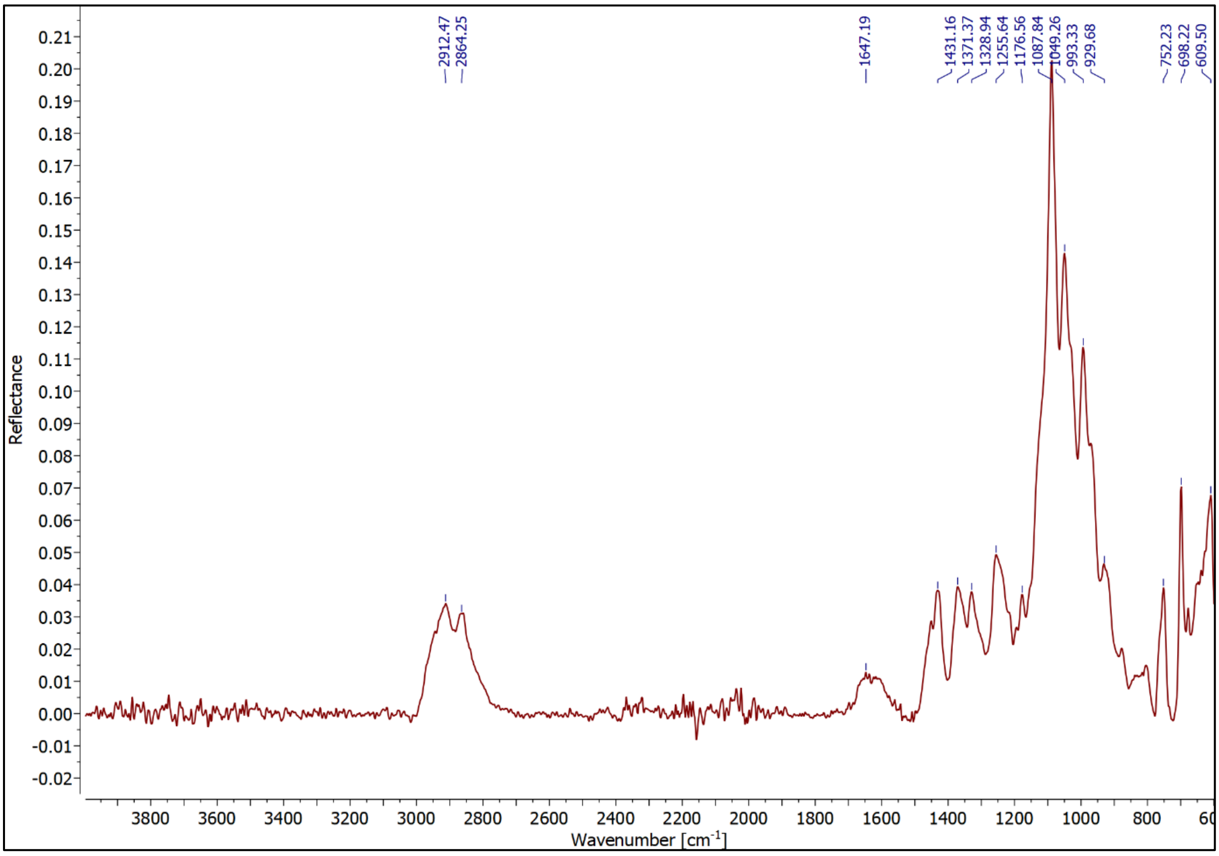

Figure S12. FTIR (ATR) spectra of immobilized catalyst **14b**.

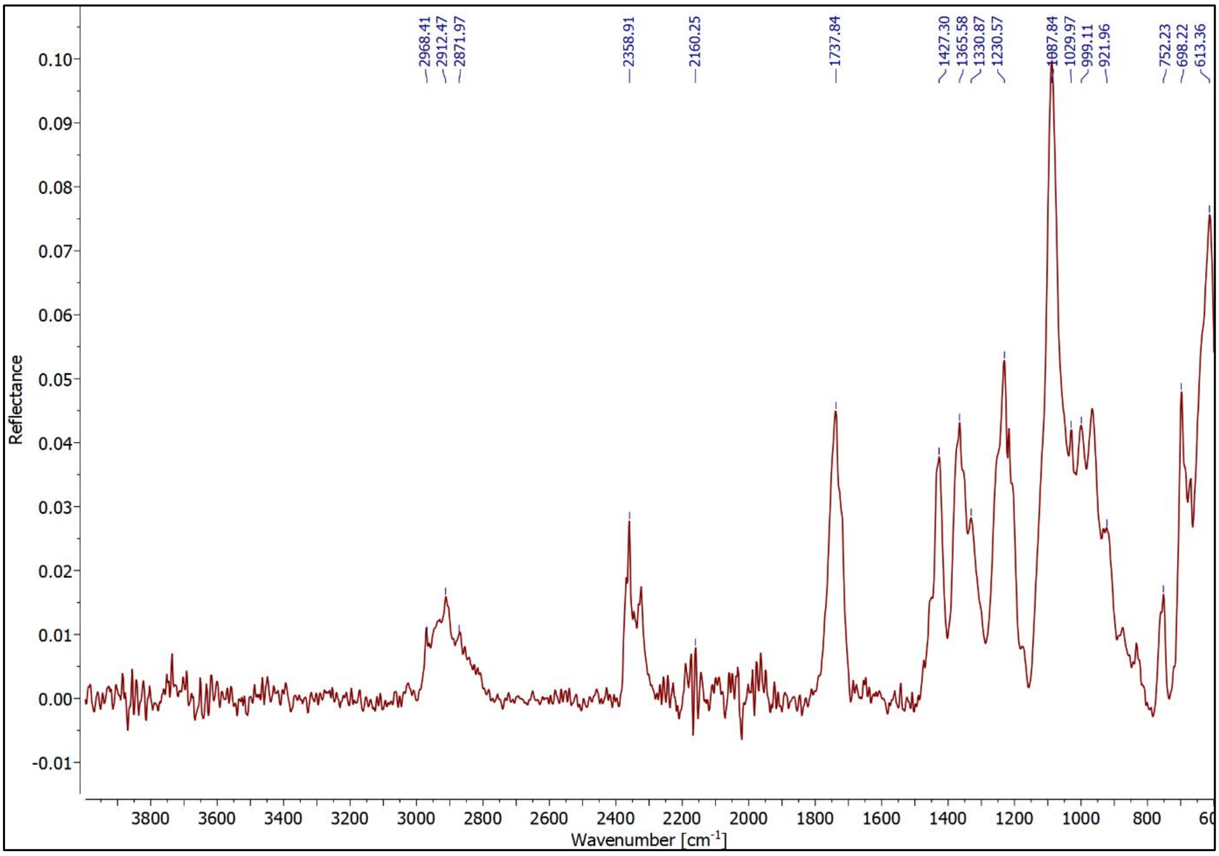

Figure S13. FTIR (ATR) spectra of immobilized catalyst **15a**.

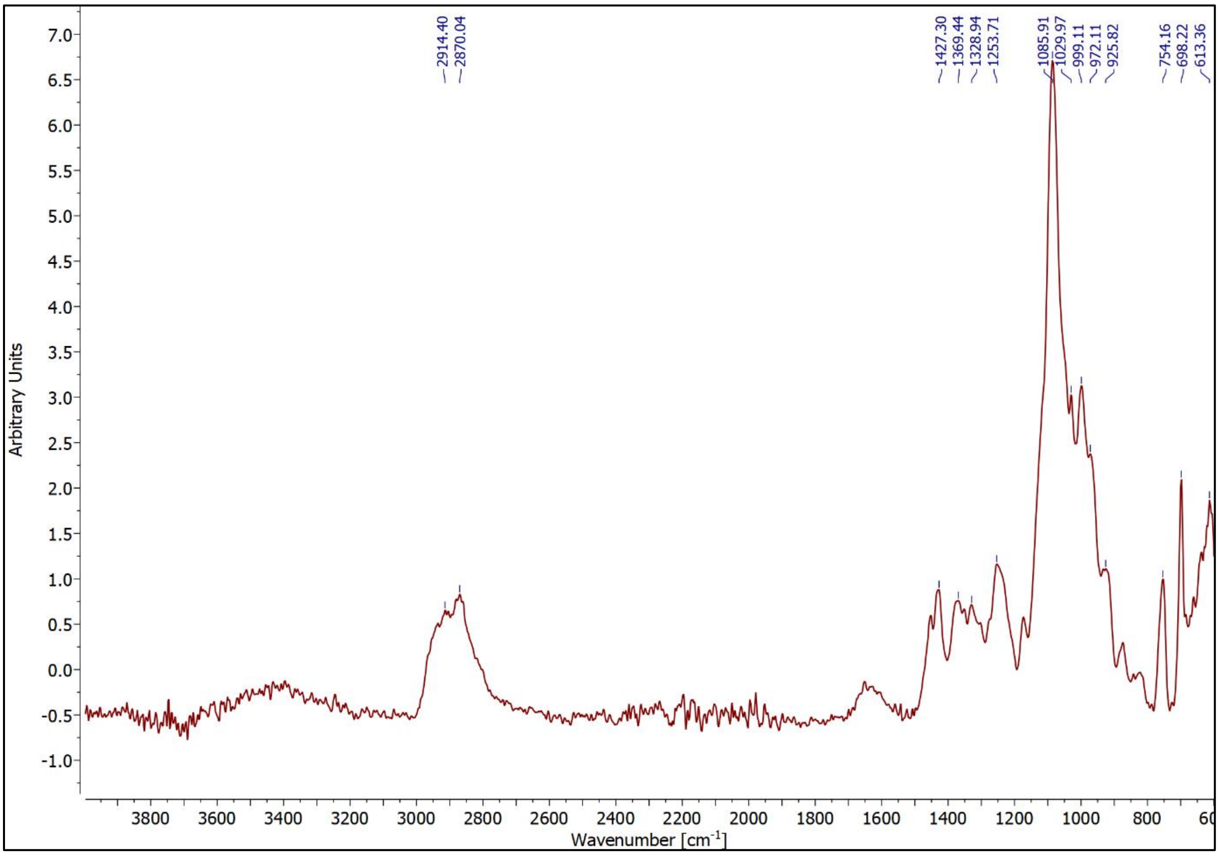

Figure S14. FTIR (ATR) spectra of immobilized catalyst **15b**.

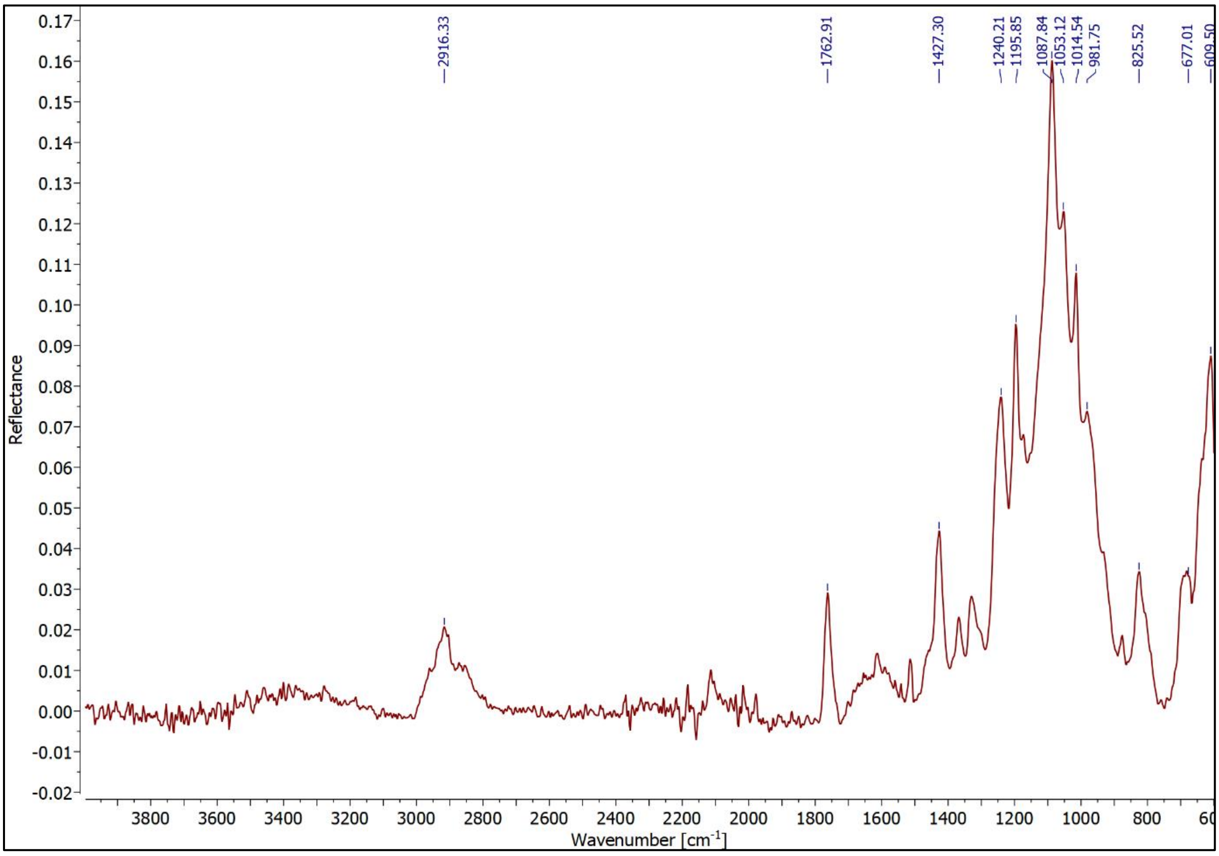

Figure S15. FTIR (ATR) spectra of immobilized catalyst **16a**.

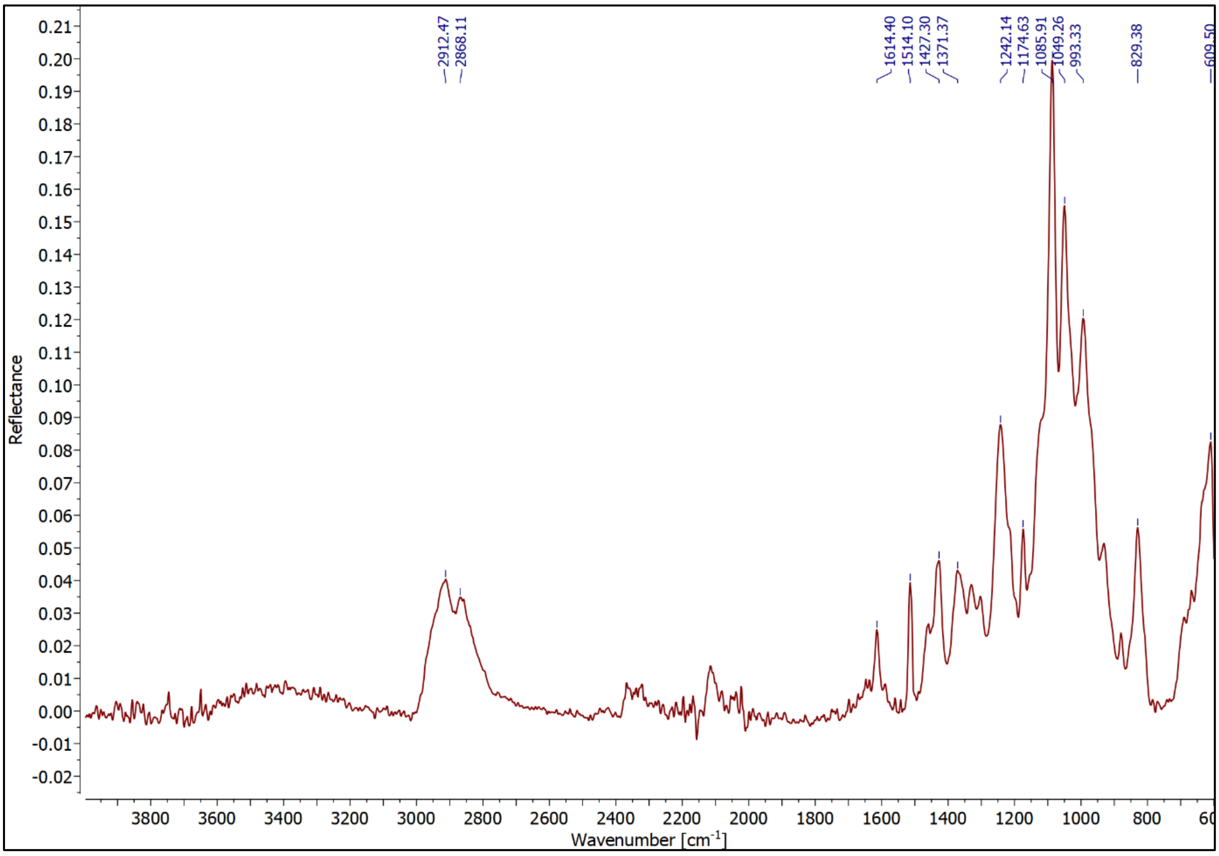

Figure S16. FTIR (ATR) spectra of immobilized catalyst **16b**.

Compound: **Catalyst:**

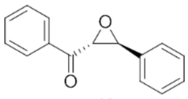

19

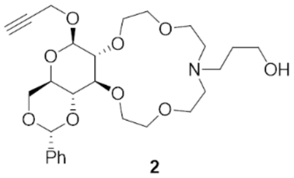

2

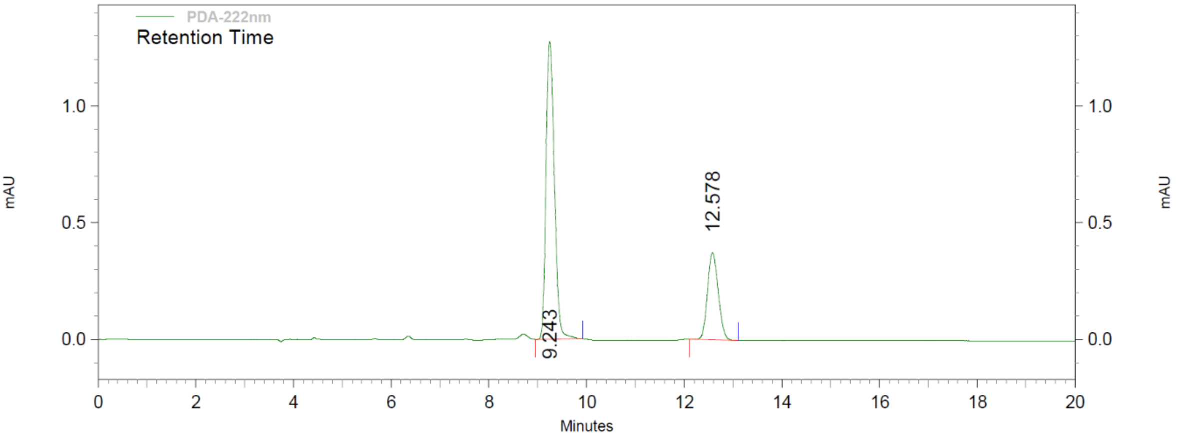

PDA-222nm Results (System (2022.11.09. 10:39:02) (Reprocessed))

Name	Retention Time	Area	Area Percent	Integration Codes
	9.243	15165707	72.795	MM
	12.578	5667701	27.205	MM
Totals		20833408	100.000	

Figure S17. Chiral HPLC chromatogram of **19** (Scheme 5, catalyst **2**).

Compound:

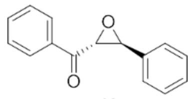

19

Catalyst:

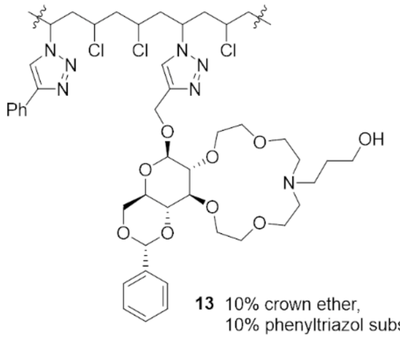

13 10% crown ether,
10% phenyltriazol substitution

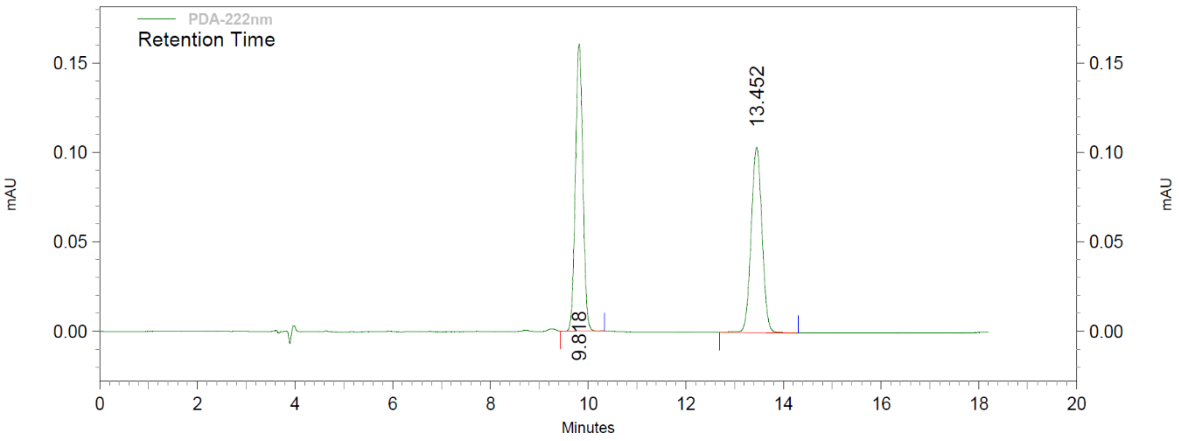

PDA-222nm Results (System (2022.10.10. 9:06:53) (Reprocessed) (Aborted Run))

Name	Retention Time	Area	Area Percent	Integration Codes
	9.818	1698295	51.441	MM
	13.452	1603167	48.559	MM

Totals		3301462	100.000	

Figure S18. Chiral HPLC chromatogram of **19** (Scheme 5, catalyst **13**).

Compound: **Catalyst:**

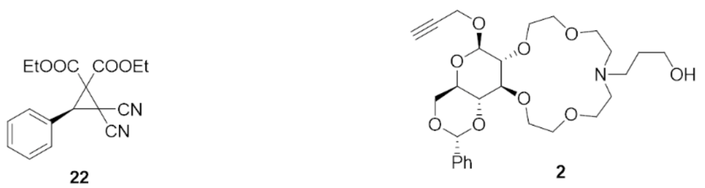

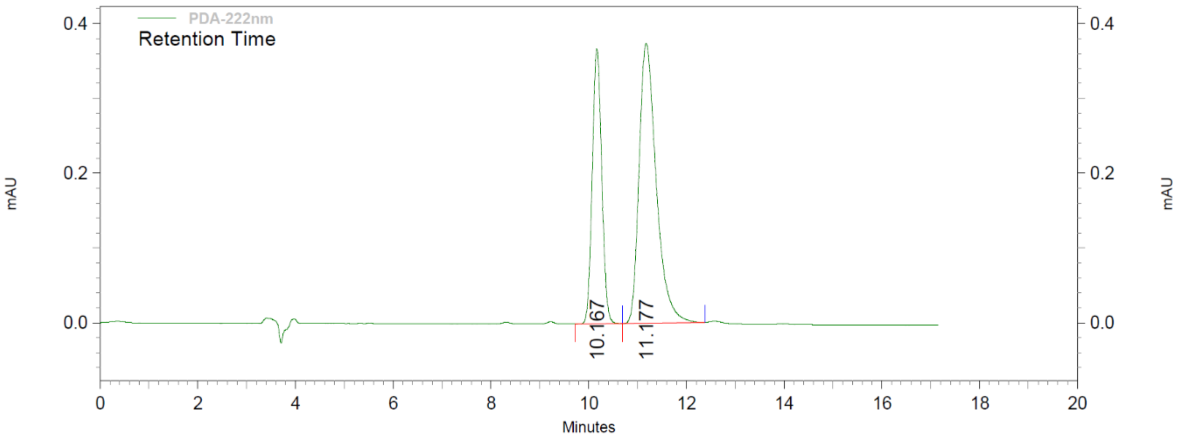

PDA-222nm Results (System (2022.11.09. 10:06:03) (Reprocessed) (Aborted Run))

Name	Retention Time	Area	Area Percent	Integration Codes
	10.167	4968382	35.250	Mx
	11.177	9126192	64.750	xM
Totals		14094574	100.000	

Figure S19. Chiral HPLC chromatogram of **22** (Scheme 6, catalyst **2**).

Compound:

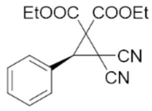

22

Catalyst:

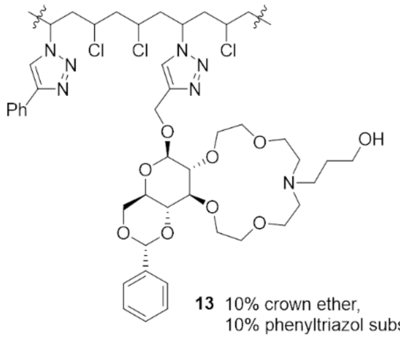

13 10% crown ether,
10% phenyltriazol substitution

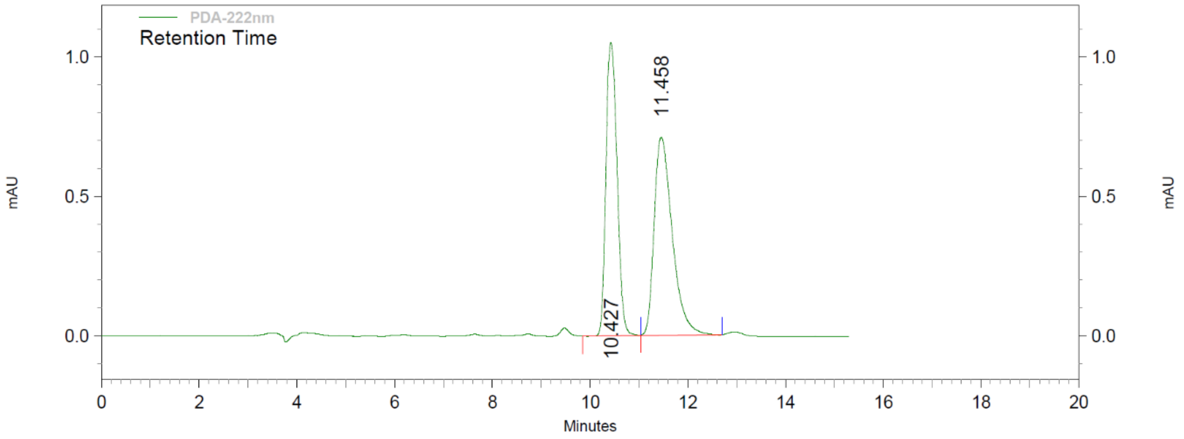

PDA-222nm
Results (System
(2022.10.10.
11:00:26)
(Reprocessed)
(Aborted Run))

Name	Retention Time	Area	Area Percent	Integration Codes
	10.427	16585295	47.545	Mx
	11.458	18298234	52.455	xM
Totals		34883529	100.000	

Figure S20. Chiral HPLC chromatogram of **22** (Scheme 6, catalyst **13**).

Compound: **Catalyst:**

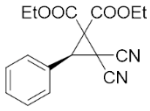

22

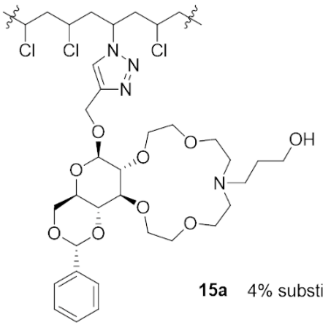

15a 4% substitution

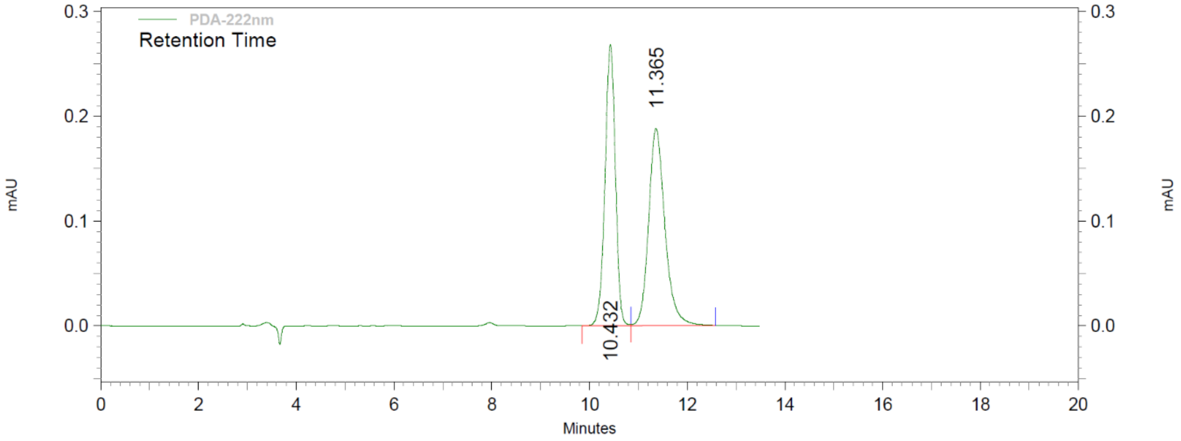

PDA-222nm
Results (System
(2023.01.11.
10:36:59)
(Reprocessed)
(Aborted Run))

Name	Retention Time	Area	Area Percent	Integration Codes
	10.432	3951740	47.465	Mx
	11.365	4373844	52.535	xM
Totals		8325584	100.000	

Figure S21. Chiral HPLC chromatogram of **22** (Scheme 6, catalyst **15a**).

Compound: **Catalyst:** **0.33 M**

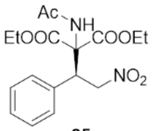

25

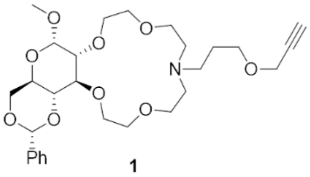

1

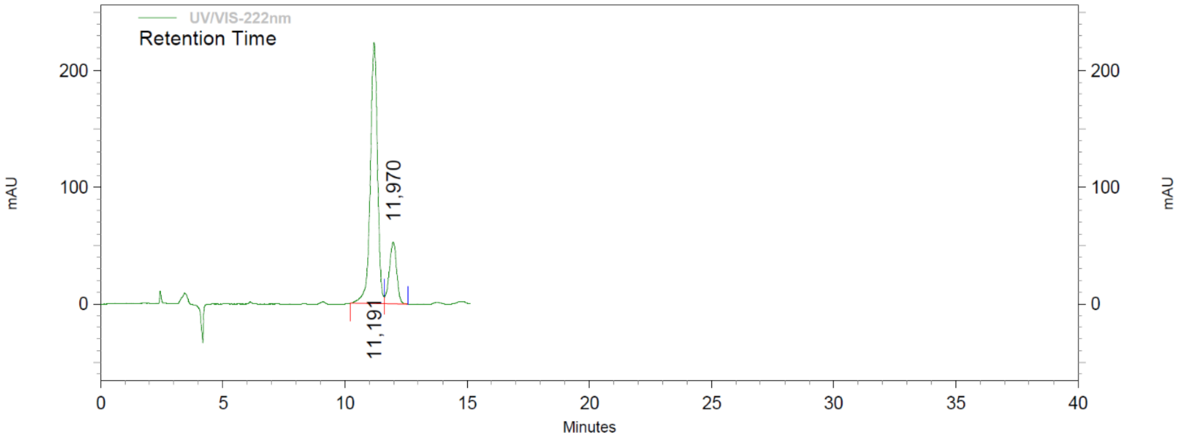

UV/VIS-222nm Results (System (2023.05.19. 14:06:00) (Reprocessed) (Aborted Run))

Name	Retention Time	Area	Area Percent	Integration Codes
	11,191	4580746	80,517	Mx
	11,970	1108404	19,483	xM
Totals		5689150	100,000	

Figure S22. Chiral HPLC chromatogram of **25** (Table 1, Entry 1).

Compound: **Catalyst:** **0.33 M**

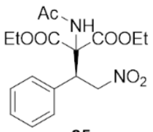

25

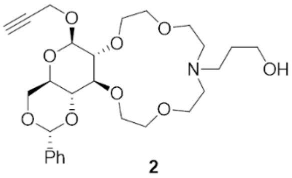

2

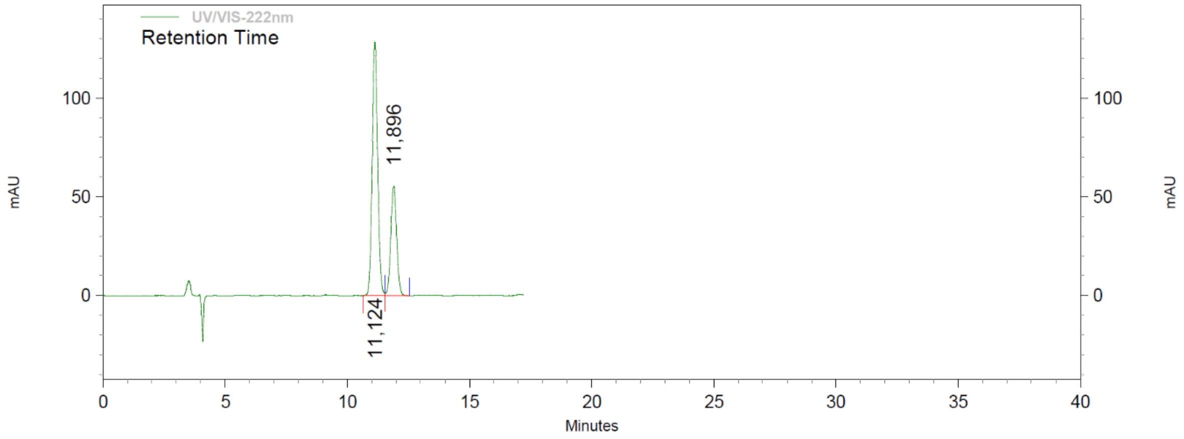

UV/VIS-222nm Results (System (2023.09.27. 11:51:39) (Reprocessed) (Aborted Run))

Name	Retention Time	Area	Area Percent	Integration Codes
	11,124	2001085	68,435	Mx
	11,896	922983	31,565	xM
Totals		2924068	100,000	

Figure S23. Chiral HPLC chromatogram of **25** (Table 1, Entry 2).

Compound: **Catalyst:** **0.33 M**

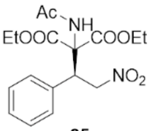

25

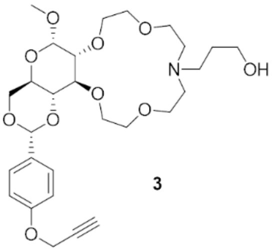

3

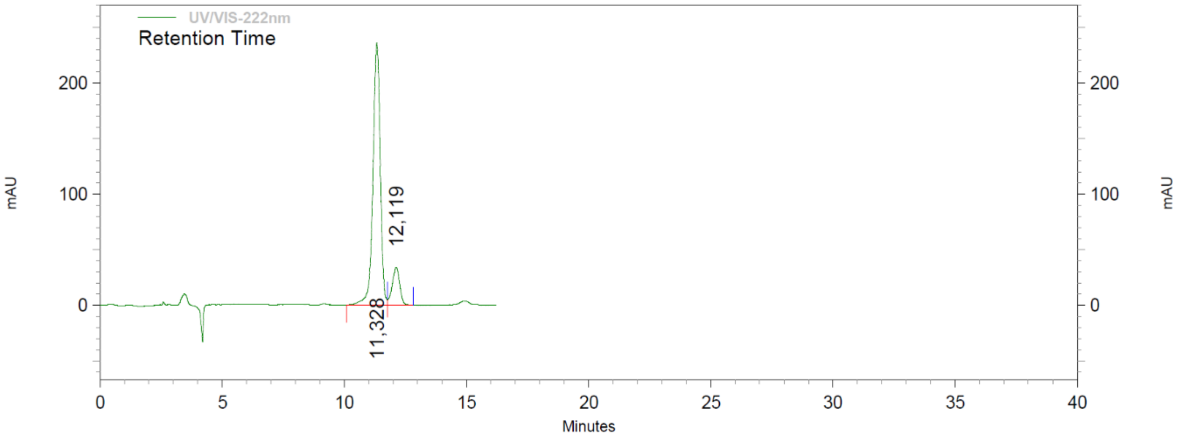

UV/VIS-222nm Results (System (2023.05.19. 14:06:51) (Reprocessed) (Aborted Run))

Name	Retention Time	Area	Area Percent	Integration Codes
	11,328	4918235	87,048	Mx
	12,119	731816	12,952	xM
Totals		5650051	100,000	

Figure S24. Chiral HPLC chromatogram of **25** (Table 1, Entry 3).

Compound: **Catalyst:** **0.1 M**

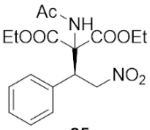

25

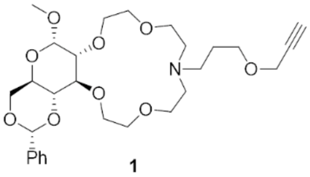

1

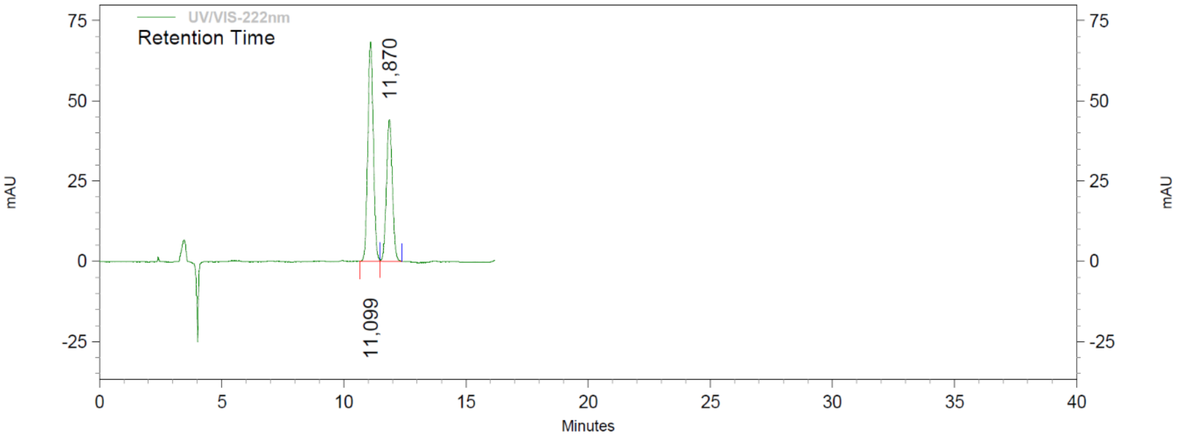

UV/VIS-222nm Results (System (2023.10.25. 13:23:47) (Reprocessed) (Aborted Run))

Name	Retention Time	Area	Area Percent	Integration Codes
	11,099	1071045	59,020	Mx
	11,870	743668	40,980	xM
Totals		1814713	100,000	

Figure S25. Chiral HPLC chromatogram of **25** (Table 1, Entry 4).

Compound: **Catalyst:** **0.1 M**

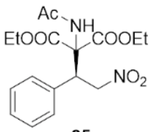

25

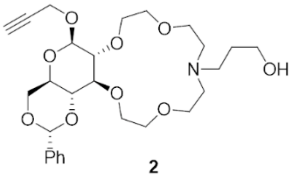

2

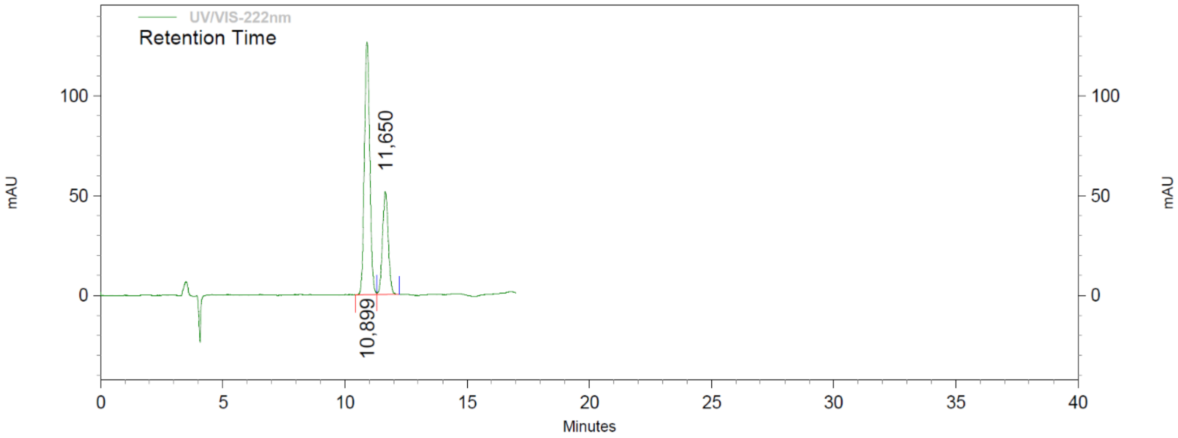

UV/VIS-222nm Results (System (2023.09.27. 11:48:08) (Reprocessed) (Aborted Run))

Name	Retention Time	Area	Area Percent	Integration Codes
	10,899	1936696	69,767	Mx
	11,650	839247	30,233	xM
Totals		2775943	100,000	

Figure S26. Chiral HPLC chromatogram of **25** (Table 1, Entry 5).

Compound: **Catalyst:** **0.1 M**

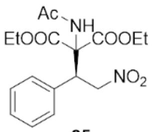

25

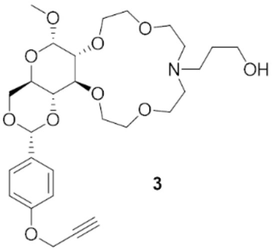

3

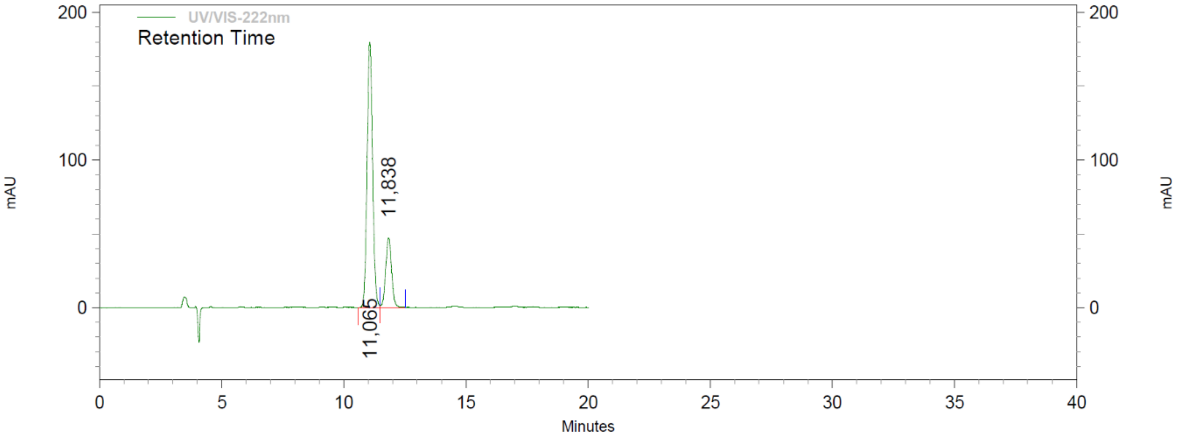

UV/VIS-222nm Results (System (2023.09.27. 11:49:03) (Reprocessed) (Aborted Run))

Name	Retention Time	Area	Area Percent	Integration Codes
	11,065	2812305	78,183	Mx
	11,838	784790	21,817	xM
Totals		3597095	100,000	

Figure S27. Chiral HPLC chromatogram of **25** (Table 1, Entry 6).

Compound:

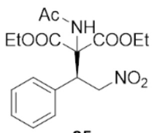

25

Catalyst:

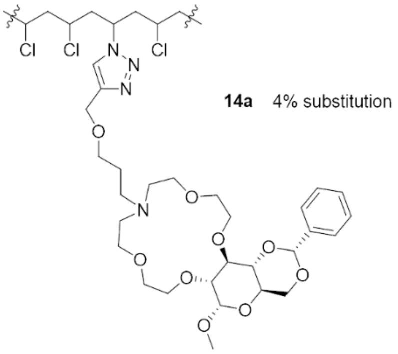

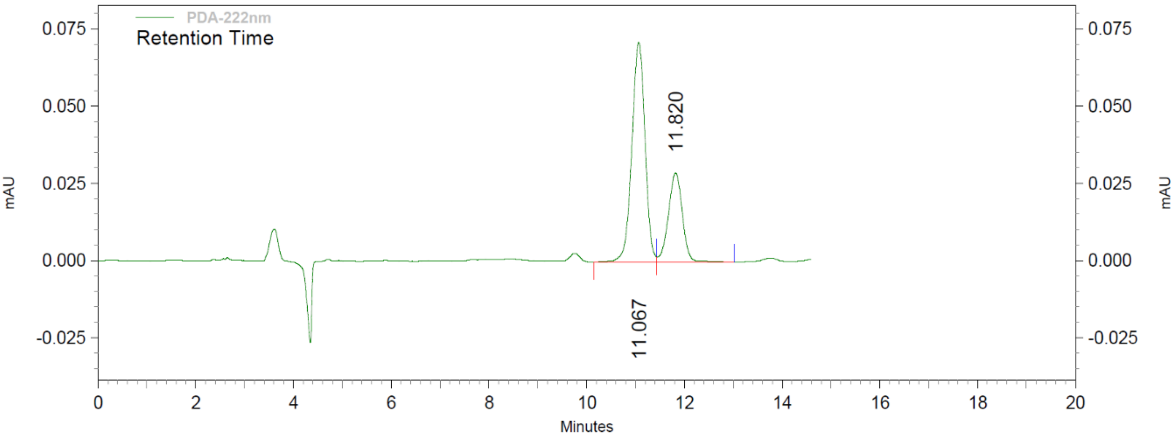

PDA-222nm
Results (System
(2023.06.26.
9:04:46)
(Reprocessed)
(Aborted Run))

Name	Retention Time	Area	Area Percent	Integration Codes
	11.067	1334072	69.877	Mx
	11.820	575111	30.123	xM
Totals		1909183	100.000	

Figure S28. Chiral HPLC chromatogram of **25** (Table 2, Entry 1).

Compound:

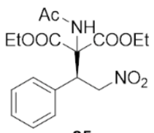

25

Catalyst:

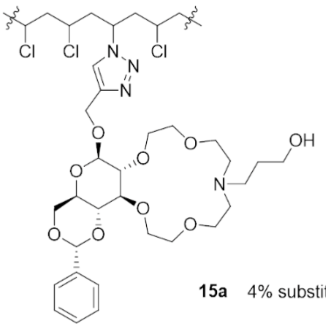

15a 4% substitution

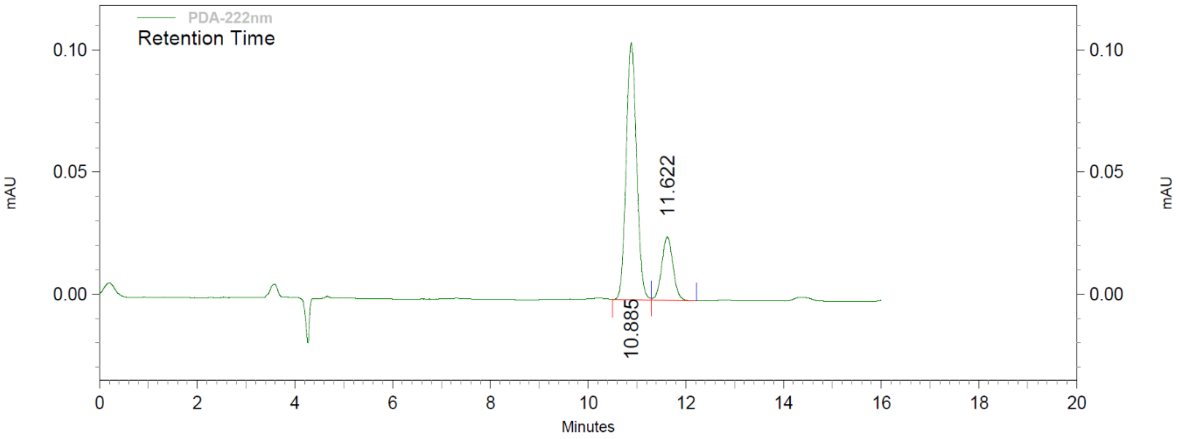

PDA-222nm
Results (System
(2023.04.24.
14:06:33)
(Reprocessed)
(Aborted Run))

Name	Retention Time	Area	Area Percent	Integration Codes
	10.885	1563386	78.953	Mx
	11.622	416754	21.047	xM
Totals		1980140	100.000	

Figure S29. Chiral HPLC chromatogram of **25** (Table 2, Entry 2).

Compound: **Catalyst:** **0.1 M, Cycle 1**

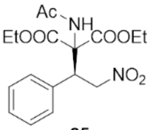

25

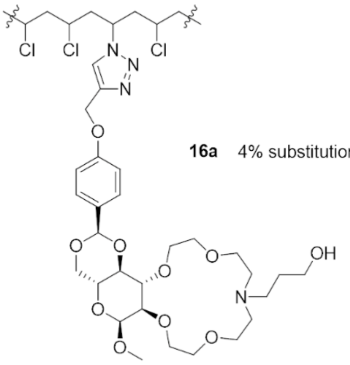

16a 4% substitution

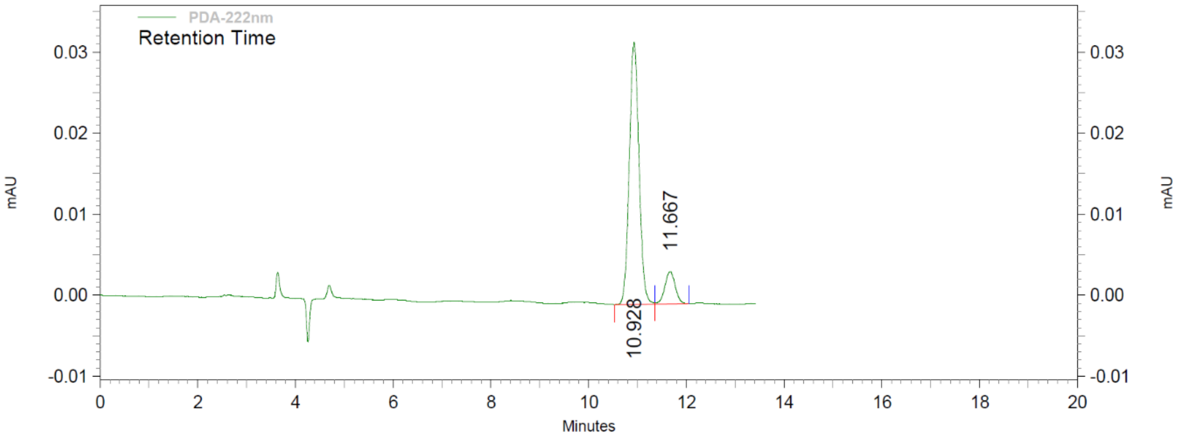

PDA-222nm
Results (System (2023.06.26. 9:03:29) (Reprocessed) (Aborted Run))

Name	Retention Time	Area	Area Percent	Integration Codes
	10.928	445630	88.482	Mx
	11.667	58009	11.518	xM
Totals		503639	100.000	

Figure S30. Chiral HPLC chromatogram of **25** (Table 2, Entry 3, Cycle 1).

Compound: **Catalyst:** **0.1 M, Cycle 2**

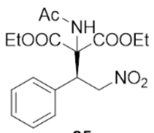

25

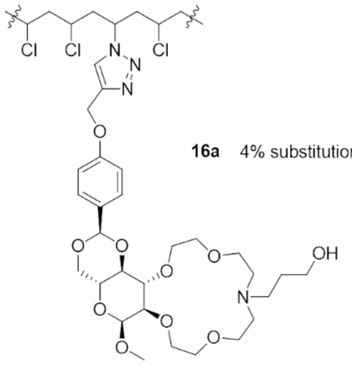

16a 4% substitution

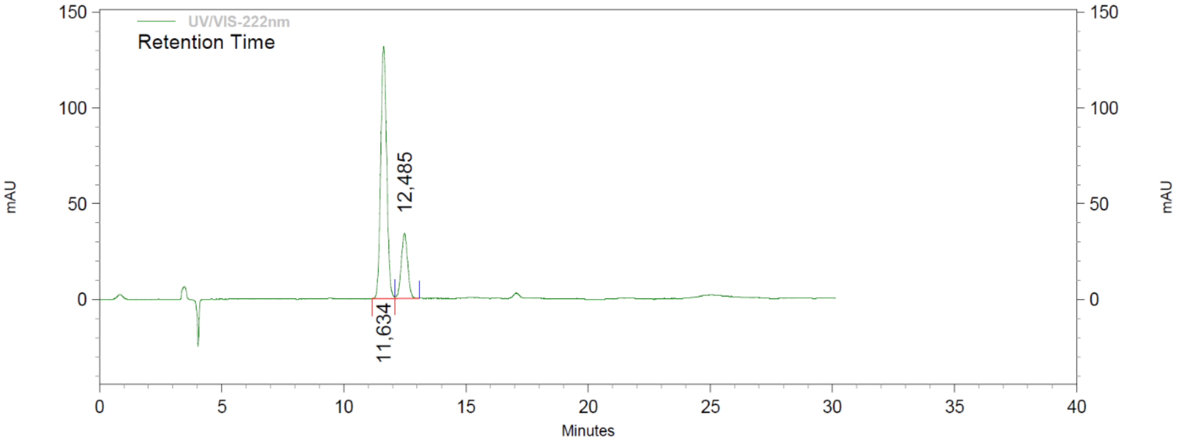

UV/VIS-222nm Results (System (2023.10.25. 13:53:03) (Reprocessed) (Aborted Run))

Name	Retention Time	Area	Area Percent	Integration Codes
	11,634	2232750	78,284	Mx
	12,485	619358	21,716	xM
Totals		2852108	100,000	

Figure S31. Chiral HPLC chromatogram of **25** (Table 2, Entry 3, Cycle 2).

Compound: **Catalyst:** **0.1 M, Cycle 3**

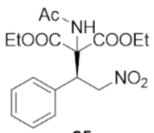

25

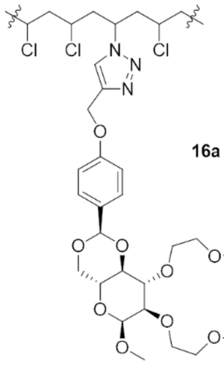

16a 4% substitution

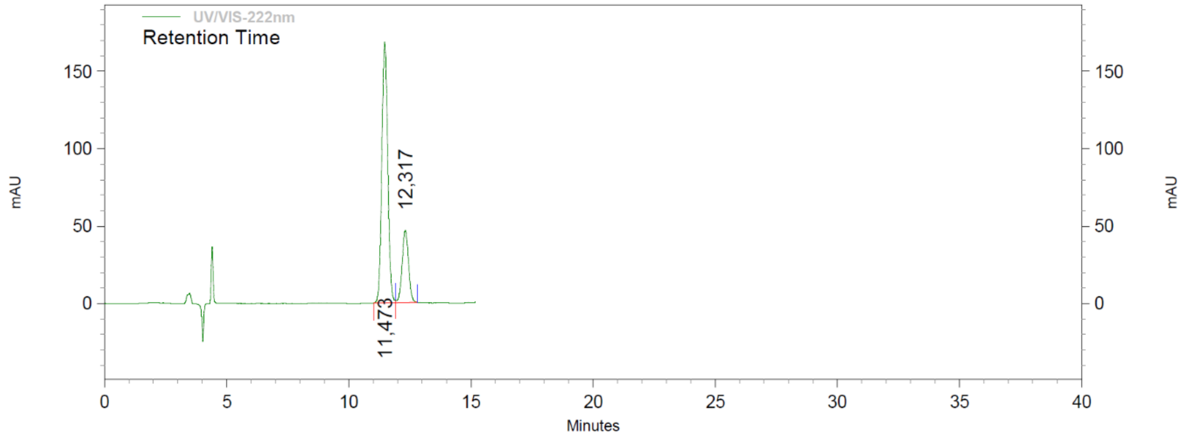

UV/VIS-222nm Results (System (2023.10.25. 13:51:56) (Reprocessed) (Aborted Run))

Name	Retention Time	Area	Area Percent	Integration Codes
	11,473	2840391	77,101	Mx
	12,317	843617	22,899	xM
Totals		3684008	100,000	

Figure S32. Chiral HPLC chromatogram of **25** (Table 2, Entry 3, Cycle 3).

Compound: **Catalyst:** **0.1 M, Cycle 4**

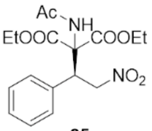

25

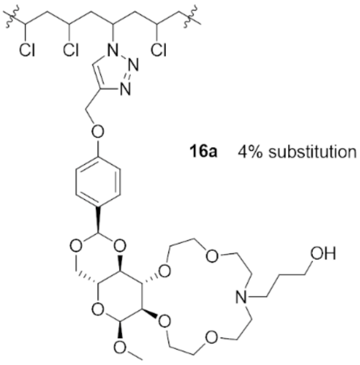

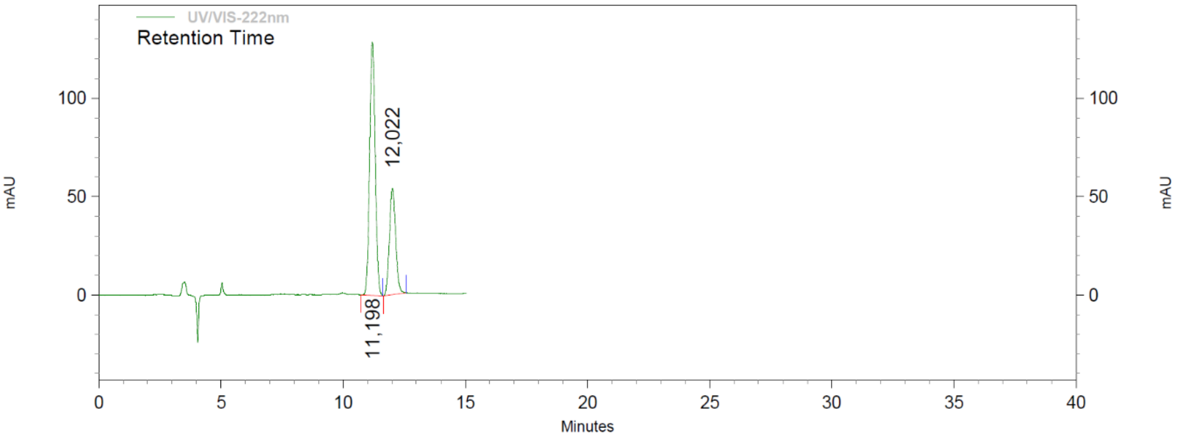

UV/VIS-222nm Results (System (2023.10.31. 12:45:19) (Reprocessed) (Aborted Run))

Name	Retention Time	Area	Area Percent	Integration Codes
	11,198	2052156	68,175	MM
	12,022	957959	31,825	MM
Totals		3010115	100,000	

Figure S33. Chiral HPLC chromatogram of **25** (Table 2, Entry 3, Cycle 4).

Compound: **Catalyst:** **0.1 M, Cycle 5**

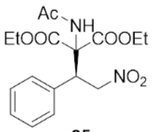

25

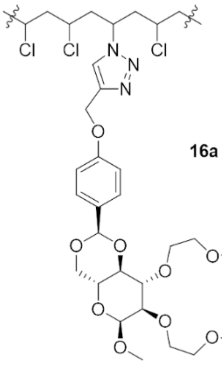

16a 4% substitution

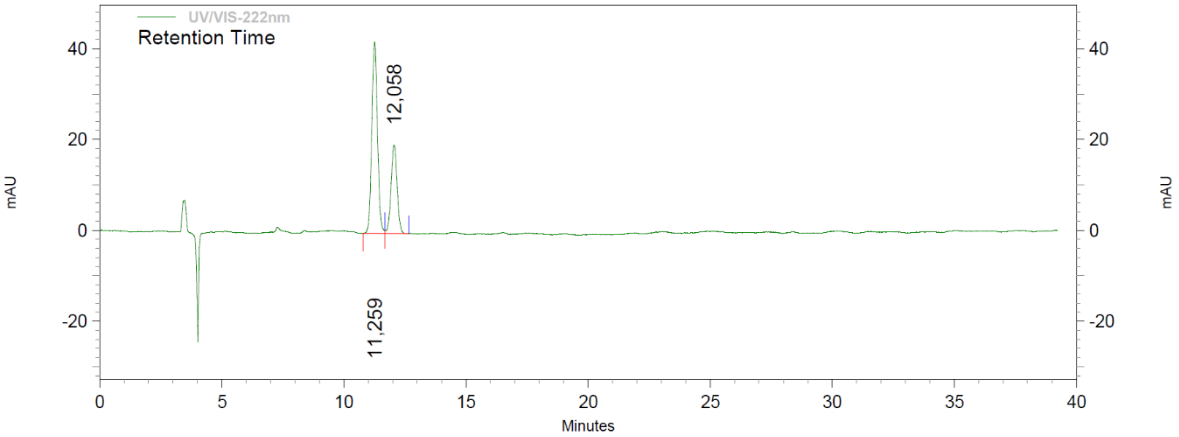

UV/VIS-222nm Results (System (2023.10.31. 12:33:50) (Reprocessed) (Aborted Run))

Name	Retention Time	Area	Area Percent	Integration Codes
	11,259	680371	66,891	Mx
	12,058	336757	33,109	xM
Totals		1017128	100,000	

Figure S34. Chiral HPLC chromatogram of **25** (Table 2, Entry 3, Cycle 5).

Compound: **Catalyst:** **0.33 M, Cycle 1**

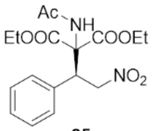

25

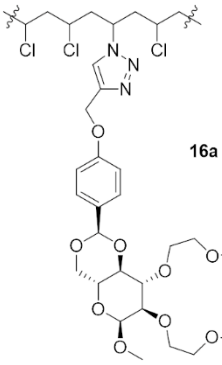

16a 4% substitution

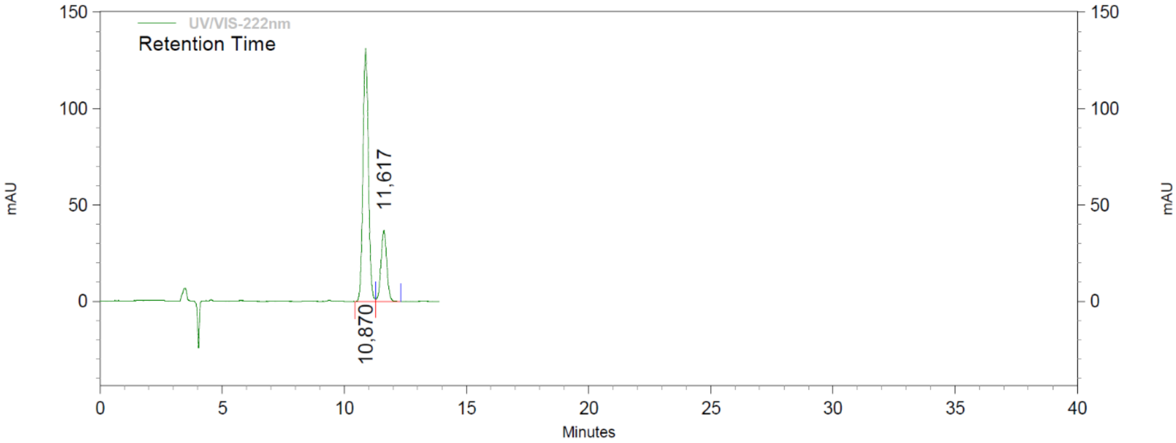

UV/VIS-222nm Results (System (2023.10.25. 13:22:51) (Reprocessed) (Aborted Run))

Name	Retention Time	Area	Area Percent	Integration Codes
	10,870	2019801	76,921	Mx
	11,617	606015	23,079	xM
Totals		2625816	100,000	

Figure S35. Chiral HPLC chromatogram of **25** (Table 2, Entry 4, Cycle 1).

Compound: **Catalyst:** **0.33 M, Cycle 2**

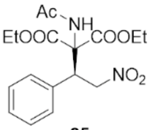

25

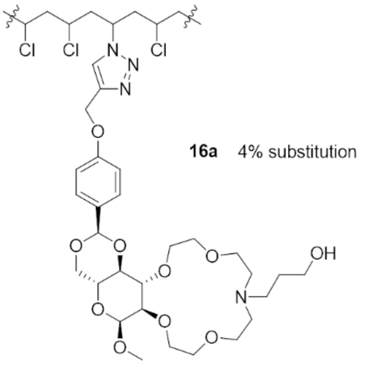

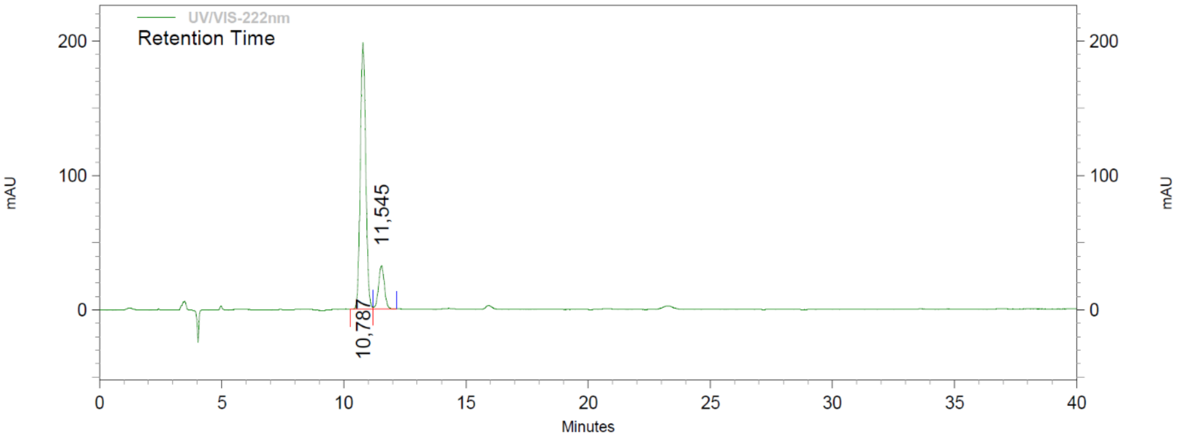

UV/VIS-222nm Results (System (2023.10.25. 13:21:17) (Reprocessed))

Name	Retention Time	Area	Area Percent	Integration Codes
	10,787	3088201	85,150	Mx
	11,545	538565	14,850	xM
Totals		3626766	100,000	

Figure S36. Chiral HPLC chromatogram of **25** (Table 2, Entry 4, Cycle 2).

Compound: **Catalyst:** **0.33 M, Cycle 3**

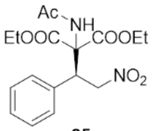

25

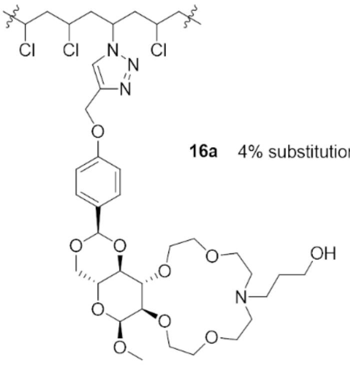

16a 4% substitution

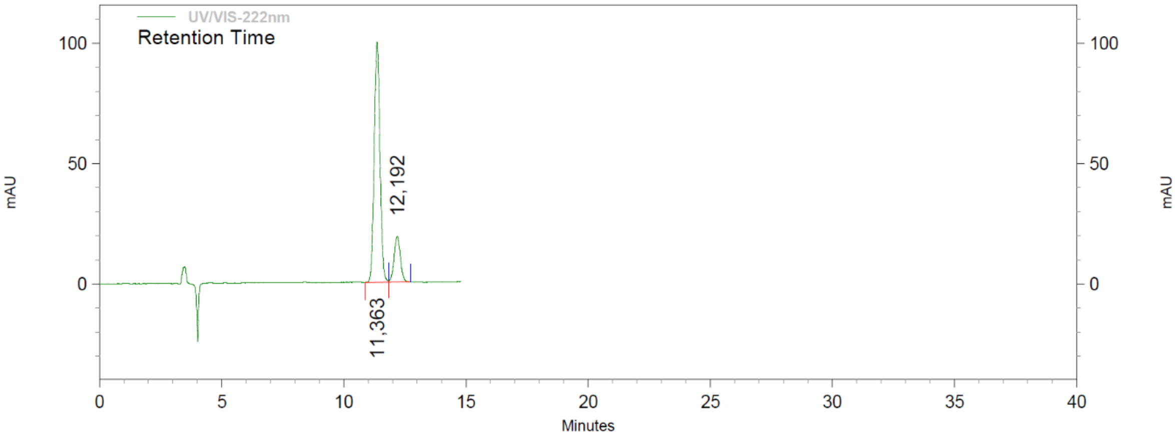

UV/VIS-222nm Results (System (2023.10.31. 12:35:14) (Reprocessed) (Aborted Run))

Name	Retention Time	Area	Area Percent	Integration Codes
	11,363	1645091	82,941	Mx
	12,192	338355	17,059	xM
Totals		1983446	100,000	

Figure S37. Chiral HPLC chromatogram of **25** (Table 2, Entry 4, Cycle 3).

Compound: **Catalyst:** **0.33 M, Cycle 4**

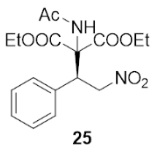

25

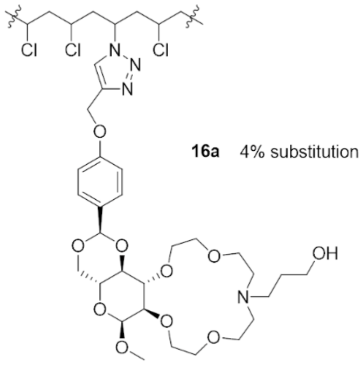

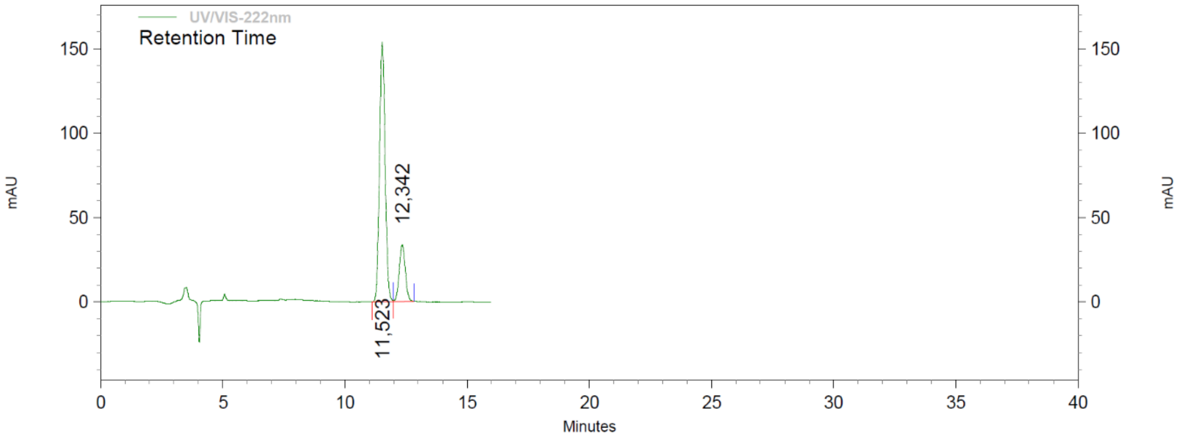

UV/VIS-222nm Results (System (2023.10.31. 12:36:29) (Reprocessed) (Aborted Run))

Name	Retention Time	Area	Area Percent	Integration Codes
	11,523	2474920	81,115	Mx
	12,342	576192	18,885	xM
Totals		3051112	100,000	

Figure S38. Chiral HPLC chromatogram of **25** (Table 2, Entry 4, Cycle 4).

Compound: **Catalyst:** **0.33 M, Cycle 5**

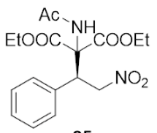

25

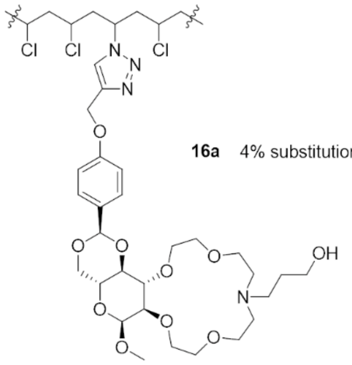

16a 4% substitution

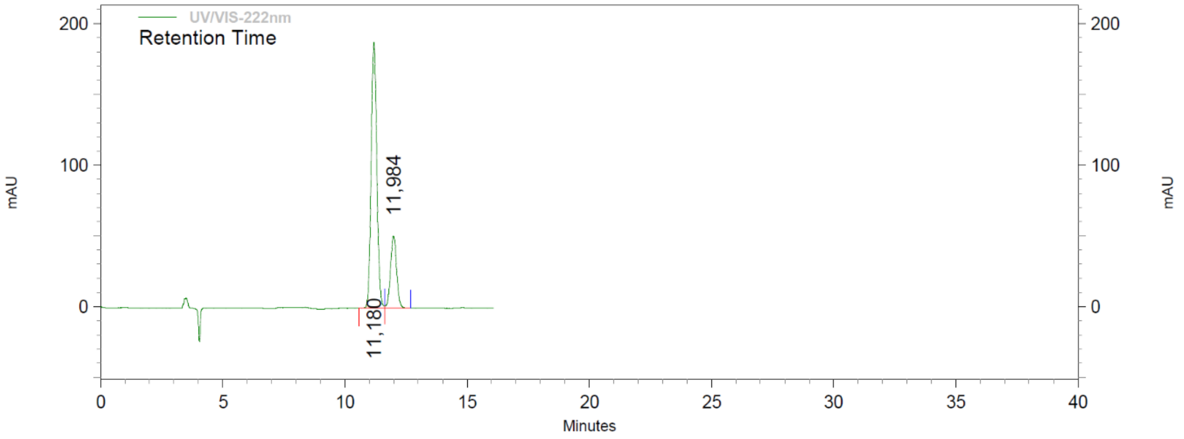

UV/VIS-222nm Results (System (2023.10.31. 12:37:46) (Reprocessed) (Aborted Run))

Name	Retention Time	Area	Area Percent	Integration Codes
	11,180	3020925	77,483	Mx
	11,984	877907	22,517	xM
Totals		3898832	100,000	

Figure S39. Chiral HPLC chromatogram of **25** (Table 2, Entry 4, Cycle 5).

Compound: **Catalyst:** **Cycle 1**

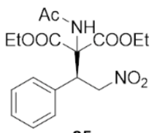

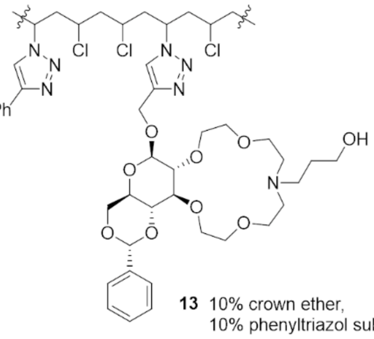

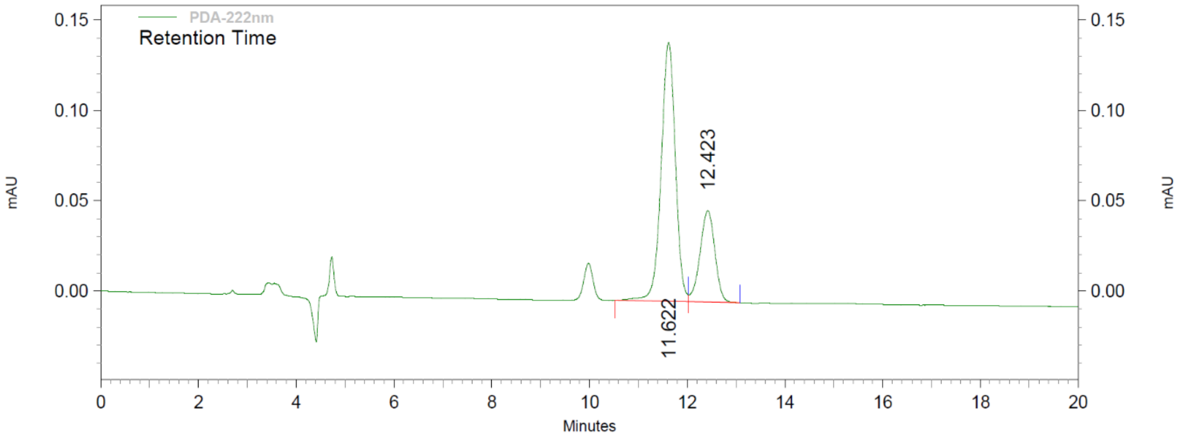

PDA-222nm
Results (System
(2023.03.02.
10:45:12)
(Reprocessed))

Name	Retention Time	Area	Area Percent	Integration Codes
	11.622	2911722	73.143	Mx
	12.423	1069165	26.857	xM

Totals				
		3980887	100.000	

Figure S40. Chiral HPLC chromatogram of **25** (Table 2, Entry 5, Cycle 1).

Compound: **Catalyst:** **Cycle 2**

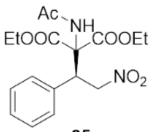

25

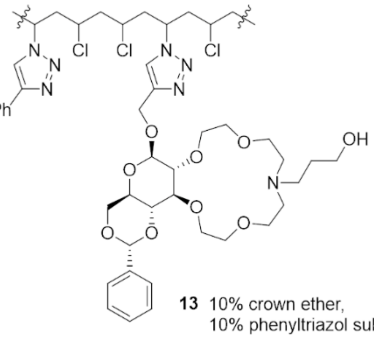

13 10% crown ether,
10% phenyltriazol substitution

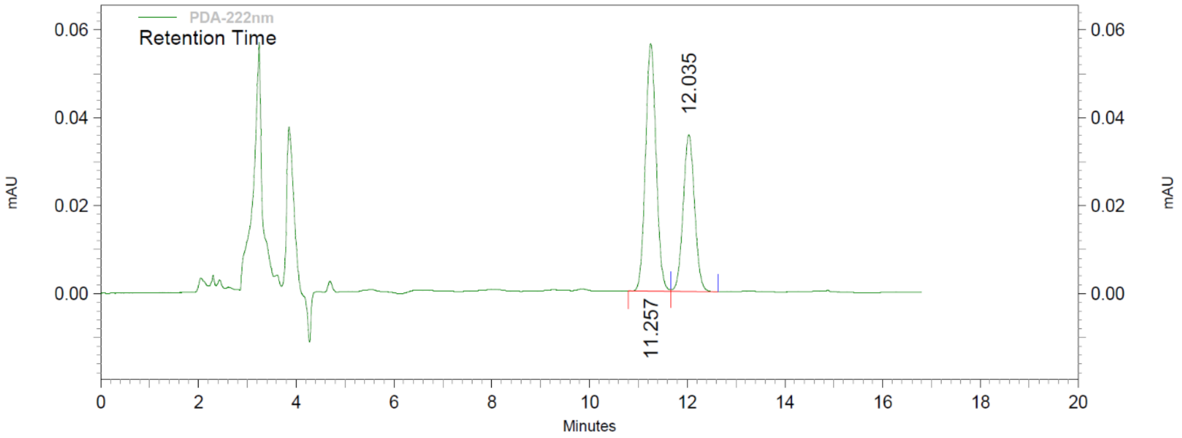

PDA-222nm
Results (System
(2023.04.18.
10:07:09)
(Reprocessed)
(Aborted Run))

Name	Retention Time	Area	Area Percent	Integration Codes
	11.257	852419	59.466	Mx
	12.035	581048	40.534	xM

Totals				
		1433467	100.000	

Figure S41. Chiral HPLC chromatogram of **25** (Table 2, Entry 5, Cycle 2).

Compound: **Catalyst:** **Cycle 3**

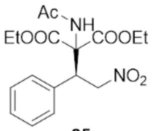

25

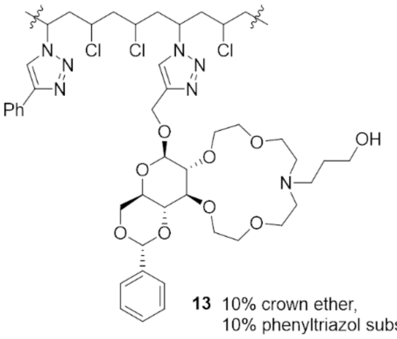

13 10% crown ether,
10% phenyltriazol substitution

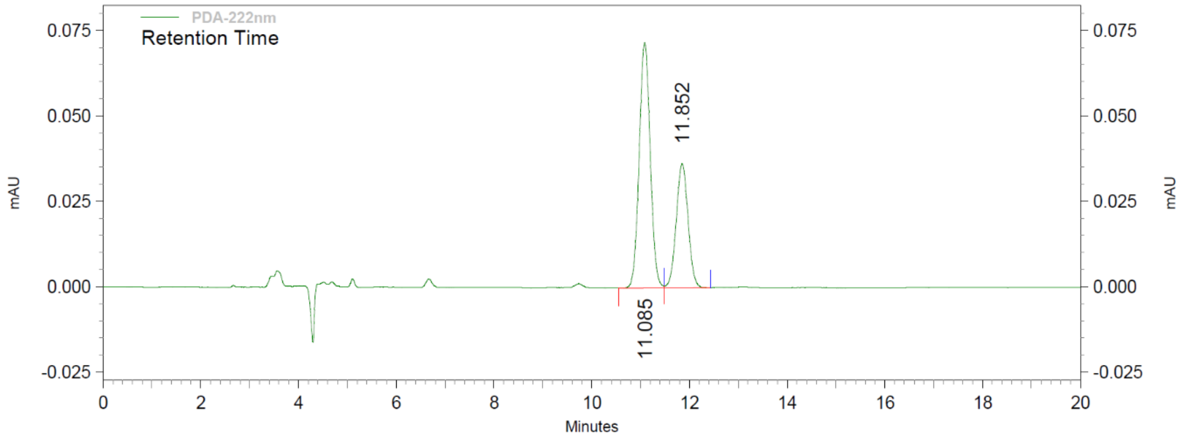

PDA-222nm
Results (System
(2023.04.18.
10:08:29)
(Reprocessed))

Name	Retention Time	Area	Area Percent	Integration Codes
	11.085	1129869	64.825	Mx
	11.852	613085	35.175	xM
Totals		1742954	100.000	

Figure S42. Chiral HPLC chromatogram of **25** (Table 2, Entry 5, Cycle 3).

Compound: **Catalyst:** **Cycle 4**

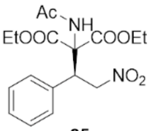

25

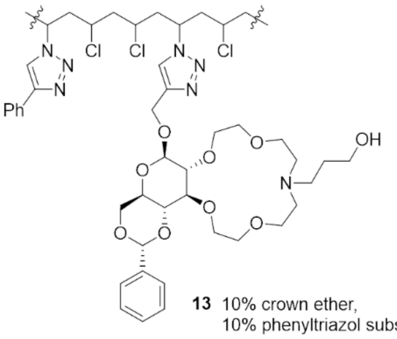

13 10% crown ether, 10% phenyltriazol substitution

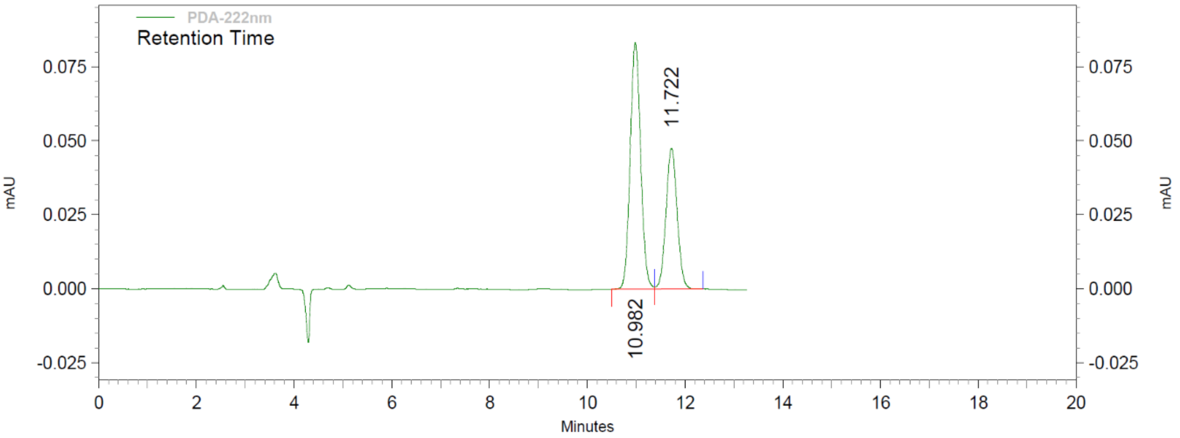

PDA-222nm Results (System (2023.04.24. 14:03:33) (Reprocessed) (Aborted Run))

Name	Retention Time	Area	Area Percent	Integration Codes
	10.982	1239455	62.128	Mx
	11.722	755554	37.872	xM
Totals		1995009	100.000	

Figure S43. Chiral HPLC chromatogram of **25** (Table 2, Entry 5, Cycle 4).

Compound:

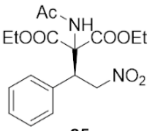

25

Catalyst:

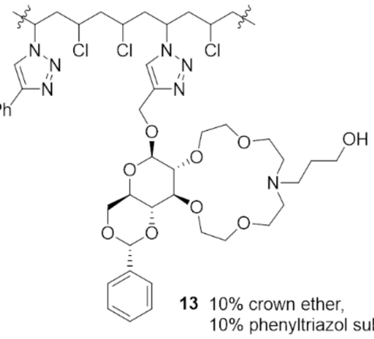

13 10% crown ether,
10% phenyltriazol substitution

Cycle 5

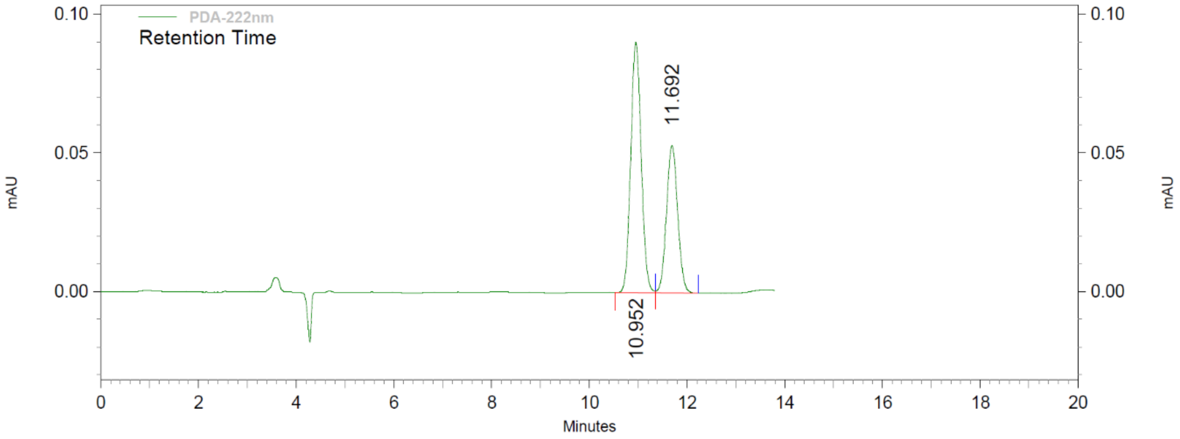

PDA-222nm
Results (System
(2023.04.24.
14:04:38)
(Reprocessed)
(Aborted Run))

Name	Retention Time	Area	Area Percent	Integration Codes
	10.952	1341447	61.339	Mx
	11.692	845502	38.661	xM
Totals		2186949	100.000	

Figure S44. Chiral HPLC chromatogram of **25** (Table 2, Entry 5, Cycle 5).

Compound:

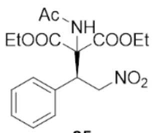

25

Catalyst:

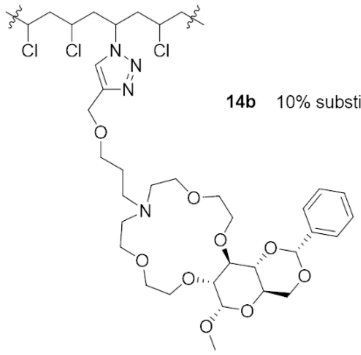

14b 10% substitution

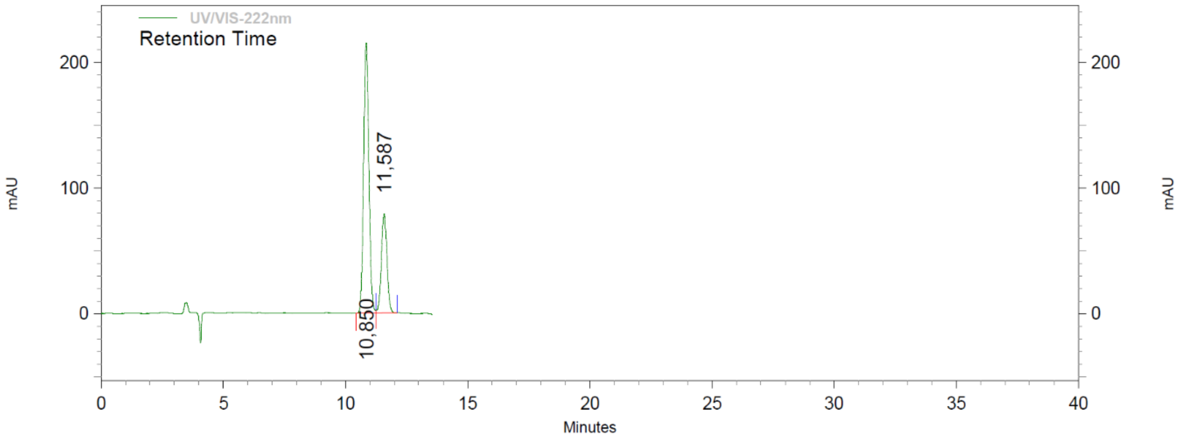

UV/VIS-222nm Results (System (2023.09.21. 12:08:55) (Reprocessed) (Aborted Run))

Name	Retention Time	Area	Area Percent	Integration Codes
	10,850	3226493	71,922	Mx
	11,587	1259588	28,078	xM
Totals		4486081	100,000	

Figure S45. Chiral HPLC chromatogram of **25** (Table 2, Entry 6).

Compound:

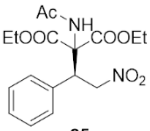

Catalyst:

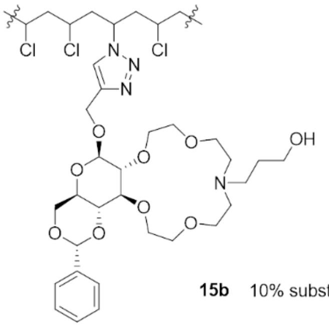

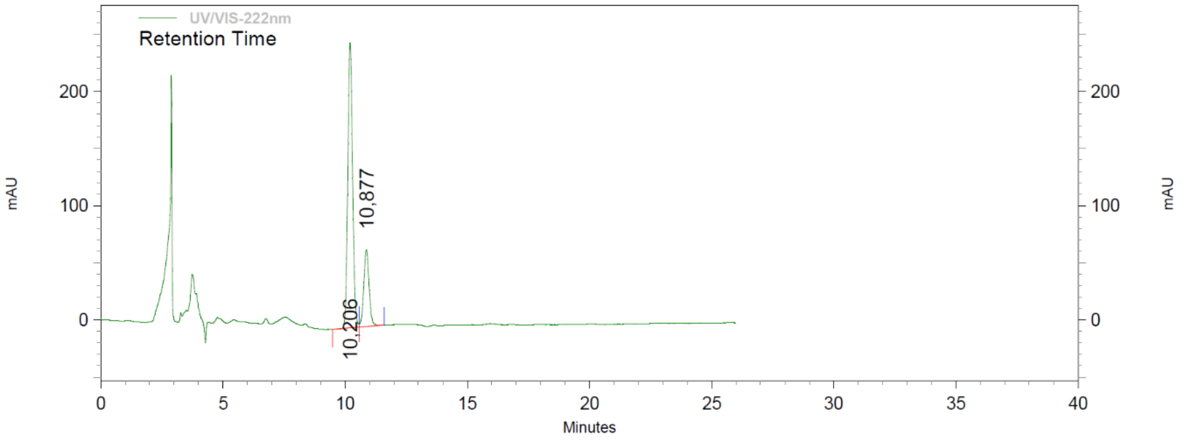

UV/VIS-222nm Results (System (2023.09.21. 12:11:14) (Reprocessed) (Aborted Run))

Name	Retention Time	Area	Area Percent	Integration Codes
	10,206	3418703	76,877	Mx
	10,877	1028258	23,123	xM
Totals		4446961	100,000	

Figure S46. Chiral HPLC chromatogram of **25** (Table 2, Entry 7).

Compound:

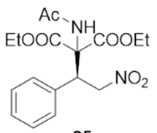

25

Catalyst:

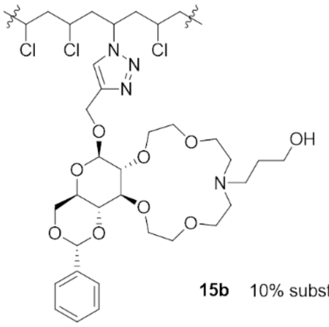

15b 10% substitution

carried out in dark

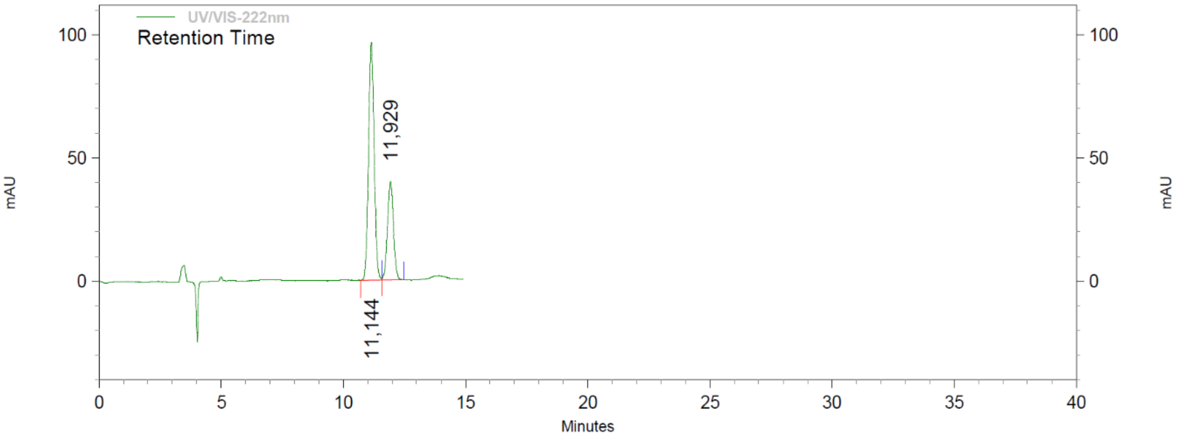

UV/VIS-222nm Results (System (2023.10.25. 13:24:53) (Reprocessed) (Aborted Run))

Name	Retention Time	Area	Area Percent	Integration Codes
	11,144	1542296	69,370	Mx
	11,929	680997	30,630	xM
Totals		2223293	100,000	

Figure S47. Chiral HPLC chromatogram of **25** (Table 2, Entry 8).

Compound: **Catalyst:**

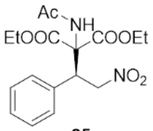

25

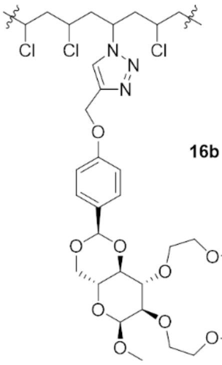

16b 10% substitution

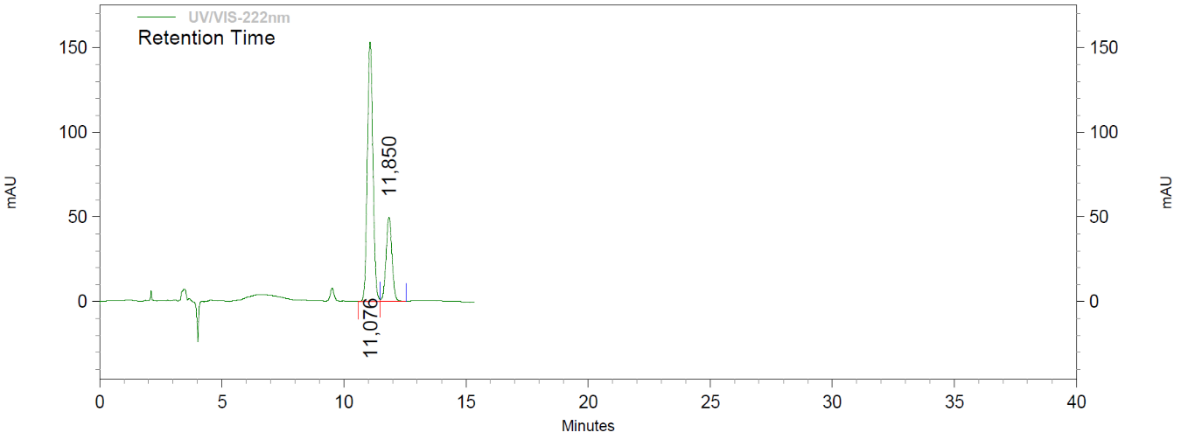

UV/VIS-222nm Results (System (2023.10.25. 13:50:46) (Reprocessed) (Aborted Run))

Name	Retention Time	Area	Area Percent	Integration Codes
	11,076	2413918	74,167	Mx
	11,850	840807	25,833	xM
Totals		3254725	100,000	

Figure S48. Chiral HPLC chromatogram of **25** (Table 2, Entry 9).
